# Comparative Efficacy and Safety of Interventions for the Treatment of Oral Lichen Planus: A Systematic Review and Network Meta-Analysis

**DOI:** 10.3390/jcm12082763

**Published:** 2023-04-07

**Authors:** Xin Yi Leong, Divya Gopinath, Sakil M. Syeed, Sajesh K. Veettil, Naresh Yedthare Shetty, Rohit Kunnath Menon

**Affiliations:** 1 School of Dentistry, International Medical University, Kuala Lumpur 57000, Malaysia; 2 Department of Basic Medical and Dental Sciences, College of Dentistry, Ajman University, Ajman P.O. Box 346, United Arab Emirates; 3 Department of Pharmacotherapy, College of Pharmacy, University of Utah, Salt Lake City, UT 84112, USA; 4 Department of Clinical Sciences, College of Dentistry, Ajman University, Ajman P.O. Box 346, United Arab Emirates

**Keywords:** systematic review, network meta-analysis, oral lichen planus, management, OLP

## Abstract

Background: This systematic review and network meta-analysis aimed to assess comparative efficacy and safety of interventions to treat symptomatic, biopsy-proven oral lichen planus (OLP). Methods: Search was conducted for trials published in Medline, Embase and Cochrane Central Register of Controlled Trials. Network meta-analysis was performed on data from randomized controlled trials that assessed efficacy and safety of interventions used in the treatment of OLP. Agents were ranked according to their effectiveness in treatment of OLP based on outcomes using surface under the cumulative ranking [SUCRA]. Results: In total, 37 articles were included in the quantitative analysis. Purslane was clinically significant and ranked first in improving clinical symptoms [RR = 4.53; 95% CI: 1.45, 14.11], followed by aloe vera [RR = 1.53; 95% CI: 1.05, 2.24], topical calcineurin [RR = 1.38; 95% CI: 1.06, 1.81] and topical corticosteroid [RR = 1.35 95% CI: 1.05, 1.73]. Topical calcineurin demonstrated the highest incidence of adverse effects [RR, 3.25 [95% CI: 1.19, 8.86. Topical corticosteroids were significant in achieving clinical improvement of OLP with RR1.37 [95% CI: 1.03, 1.81]. PDT [MD = −5.91 [95% CI: −8.15, –3.68] and showed statistically significant improvement in the clinical score for OLP. Conclusions: Purslane, aloe vera and photodynamic therapy appear promising in treatment of OLP. More high-quality trials are recommended for strengthening the evidence. Although topical calcineurin is significantly efficacious in the treatment of OLP, significant adverse effects are a concern for clinical use. Based on the current evidence, topical corticosteroids are recommended for treatment of OLP owing to their predictable safety and efficacy.

## 1. Introduction

Lichen Planus [LP] is a common chronic inflammatory disease involving both the skin and the mucous membranes of the body, including the oral cavity [1,2]. LP involving the oral mucosa is known as oral lichen planus [OLP]. It is a common autoimmune chronic inflammatory oral mucosal disorder affecting the stratified squamous epithelium by a cell-mediated immunological dysfunction [3]. OLP is more common in females between 30–60 years. The prevalence of OLP is reported to be at 1.27% [4]. Traditionally, several forms of OLP were described, such as reticular, papular, plaque, atrophic and ulcerative [erosive] form [5]. Atrophic and erosive forms of OLP usually present with burning sensation to intense pain, requiring treatment and hence are associated with difficulty in eating, swallowing and burning sensation with hot and spicy food [2,6,7,8]. However, currently, OLP is a dynamic disease, fluctuating often in distribution and extent of the lesions, clinical types, and their severity [9]. Hence, remission is rarely achieved in OLP and relapse is often seen even after treatment [6,7].

Numerous drugs have been used to treat OLP and proposed therapies given are typically symptomatic. However, evidence is inadequate to support the effectiveness of any specific treatment as being more superior than the other [10]. Although a wide range of systemic and topical therapies have been used to treat OLP, a majority of these therapies have not been evaluated in randomized controlled clinical trials [RCTs] [11]. Previous systematic reviews [12,13,14] on treatment of OLP demonstrated beneficial effects of using topical corticosteroids [TopCORT] or topical calcineurin inhibitors [TopCALN] in treatment settings. Other interventions such as aloe vera [AV] and photochemotherapy [PDT] were also tested in clinical trials. Most of the reported previous systematic reviews [12,13] have focused only on pairwise comparison of interventions. Comprehensive evidence comparing the relative efficacy and safety of all the available interventions has not been previously investigated. A network meta-analysis [NMA] allows for assessing the comparative efficacy and safety across a network of RCTs of all interventions to date through the enablement of investigations to combine both direct and indirect evidence [15]. NMA makes it possible to identify the most effective intervention for a given issue for which there are several potential solutions. Therefore, we aimed to perform a NMA to assess the comparative efficacy and safety of interventions used to treat symptomatic biopsy-proven OLP.

## 2. Materials and Methodology

This systematic review was performed with a priori published protocol [PROSPERO CRD42021256151] and was reported according to the Preferred Reporting Items for Systematic Reviews and Meta-Analysis [PRISMA] extension statement for incorporating network meta-analysis [NMA] for healthcare interventions [15].

### 2.1. Search Strategy and Study Selection

We identified relevant studies through a systematic search of Medline, Embase and Cochrane Central Register of Controlled Trials from the inception of databases to August 2022. To identify studies not captured by database searches, we manually checked the reference lists of published systematic reviews and identified articles.

Studies included were RCTs that met the following inclusion criteria.

(i) Population was patients with clinically- and histologically-proven lichen planus.
(ii) Intervention includes any form of local or systemic treatment for OLP.
(iii) Comparison is placebo, any other antifungal agent or no treatment.
(iv) Outcome.

Split mouth studies, in vitro studies, letter to editors, conference abstracts and non-English articles were excluded.

Two reviewers [L.X.Y. and RKM] independently screened titles and abstracts for eligible studies, followed by full text reading. Ineligible studies were excluded from the full text review, and the reasons for exclusion were documented. Any disagreements were resolved by consensus.

### 2.2. Outcomes of Interest

The primary outcome of interest was a clinical improvement (Thongprasom scale) of the disease. Secondary outcomes were clinical resolution, reduction in pain score (Thongprasom scale), clinical score and adverse effects.

### 2.3. Data Extraction and Quality Assessment

Data were extracted independently and in duplicates by the two reviewers into a data extraction form created following the Cochrane Handbook of Systematic Reviews of Interventions guidelines, by a consensus of all the reviewers. If multiple publications of the same trial were retrieved, only the most recent information or relevant data was included from these publications. The data from the RCTs were separated into the following sections: study characteristics, population characteristics, intervention characteristics, outcome definitions and measures. For all outcomes, we used the initial number of participants randomized to each trial arm and performed the analyses irrespective of how the authors of the original trials had analysed the data [intention-to-treat principle] [14]. The risk of bias [ROB] within each study was independently assessed by two reviewers [LYC, RKM] by using the revised Cochrane risk of bias tool [RoB 2.0] [16,17]. Disagreements were resolved by reviewers over discussion.

### 2.4. Data Synthesis and Statistical Analysis

The treatment effect was evaluated and calculated as the risk ratio [RR], along with a 95% confidence interval [CI]. A random-effects network meta-analysis [NMA] using a consistency model within a frequentist approach was applied to incorporate indirect with direct comparisons [18]. Network inconsistency assumption, which refers to a disagreement between the direct and indirect estimates, was evaluated using a global inconsistency test by fitting design-by-treatment in the inconsistency model [19,20]. For the missing data, we have followed the Cochrane assumption that data are assumed missing at random and that missing values were assumed to have a particular value, such as a poor outcome [21]. Heterogeneity was assessed by I^2^ statistics. The percentages indicate low (25%), medium (50%) and high (75%) heterogeneity [22,23]. Surface under the cumulative ranking [SUCRA] curves were estimated to rank the intervention hierarchy in the network meta-analysis [24]. Higher SUCRA scores [ranging from 0 to 1] correspond to higher ranking for clinical effectiveness [i.e., clinical resolution, clinical improvement] of OLP treatment. A comparison-adjusted funnel plot was used to examine the publication bias [24]. Stata version 15.0 [StataCorp, College Station, TX, USA] was used for statistical analysis and graph generation. To assess the robustness of primary efficacy outcome, a sensitivity analysis was performed by restricting studies with low risk of bias.

## 3. Results

### 3.1. Study Selection

**Figure 1:**
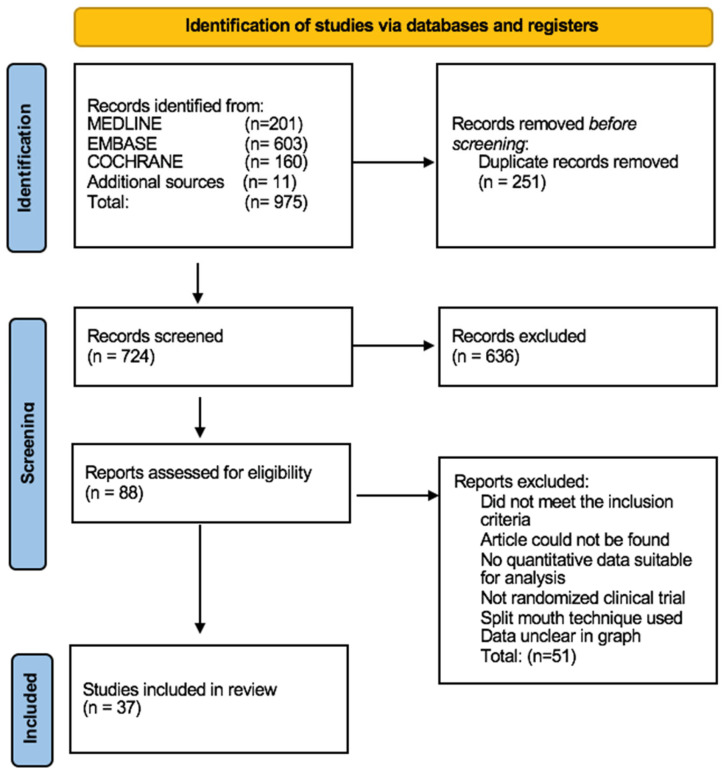
Prisma flow chart.

Our search yielded a total of 975 articles. A total of 88 articles were retained for full-text review following titles and abstracts screening and duplicate references removal. Finally, 37 articles were selected to be included in the meta-analysis. Figure 1 depicts the flow of the study selection process. The list of the excluded is provided in Supplementary Table S1.

### 3.2. Study Characteristics

Supplementary Table S2 shows the characteristics of included RCTs. The interventions assessed included amlexanox paste [AML], aloe vera [AV], curcumin gel [CUR], photodynamic therapy [PDT], placebo [PLA], purslane [PUR], systemic corticosteroid [SysCORT], topical corticosteroid [TopCORT], topical calcineurin [TopCALN] and topical calcineurin and systemic corticosteroid combined [TopCALNcoSysCORT]. Of thirty-seven included studies, five compared TopCORT and PLA [25,26,27,28,29], fifteen compared TopCORT and TopCALN [1,11,26,30,31,32,33,34,35,36,37,38,39,40,41], two compared corticosteroids with each other [39,42], six compared corticosteroids with other treatments such as PDT, AML, CUR and HA [43,44,45,46,47,48], four compared other treatments with PLA, including AV, PUR and HA [49,50,51,52], four compared TopCALN and PLA [53,54,55,56] and one compared other treatments, including aloe vera with photodynamic therapy [57].

### 3.3. Quality of RCTs

**Figure 2:**
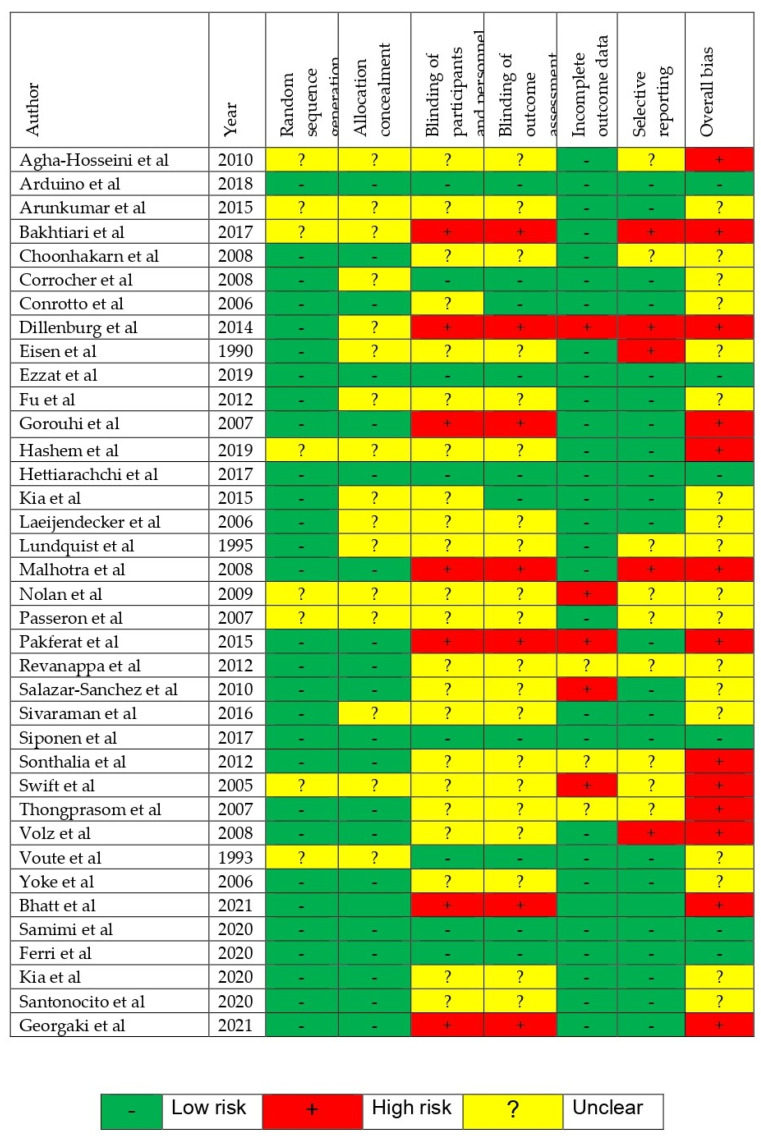
Risk of Bias [1,11,25,26,27,28,29,30,31,32,33,34,35,36,37,38,39,40,41,42,43,44,45,46,47,48,49,50,51,52,53,54,55,56,57].

Quality assessment of each study using the ROB assessment tool is provided in Figure 2. Among the RCTs, 13 trials were evaluated to be at high ROB, 6 were evaluated to be at low ROB, whereas the remaining studies were of unclear ROB. 

### 3.4. NMA Results

#### 3.4.1. Clinical Symptoms Improvement

**Figure 3:**
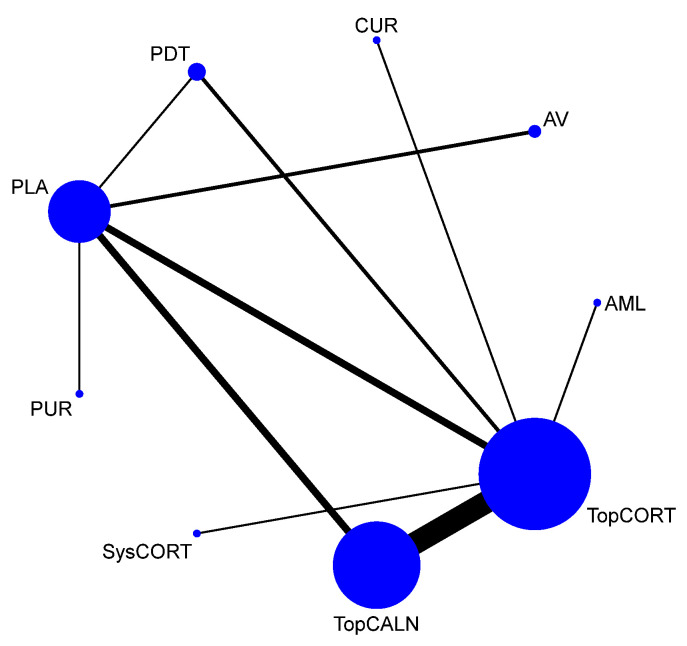
Network plot: Clinical Improvement of Oral Lichen Planus. Abbreviations: AML, amlexanox paste; AV, aloe vera; CUR, curcumin gel; PDT, photodynamic therapy; PLA, placebo; PUR, purslane; SysCORT, systemic corticosteroid; TopCALN, topical calcineurin; TopCALNcoSysCORT, topical calcineurin combined with systemic corticosteroid; TopCORT, topical corticosteroid.

**Figure 4:**
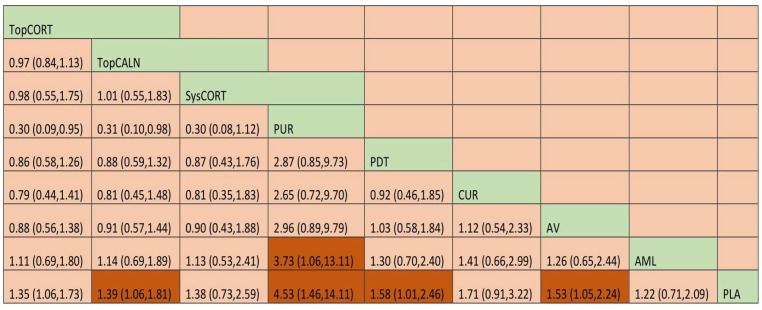
League table for the Clinical Improvement of OLP. Abbreviations: AML, amlexanox paste; AV, aloe vera; CUR, curcumin gel; PDT, photodynamic therapy; PLA, placebo; PUR, purslane; SysCORT, systemic corticosteroid; TopCALN, topical calcineurin; TopCALNcoSysCORT, topical calcineurin combined with systemic corticosteroid; TopCORT, topical corticosteroid. The green gihlights indicate the interventions and the dark highlights indicate the significant results.

A total of 37 RCTs comparing 9 interventions were included for this outcome measuring improvement in clinical symptoms. The network plot is provided in Figure 3. Network meta-analysis suggested that, compared with placebo, PUR ranked first in improving clinical symptoms [RR = 4.53; 95% CI: 1.45, 14.11, SUCRA 96.5], followed by AV [RR = 1.53; 95% CI: 1.05, 2.24, SUCRA 57.2], TopCALN [RR = 1.38; 95% CI: 1.06, 1.81, SUCRA 47.3] and TopCORT [RR = 1.35; 95% CI: 1.05, 1.73, SUCRA 40.5]. Other interventions were not statistically significant. Detailed results of SUCRA ranks and curves are presented in Supplementary Table S3 and Supplementary Figure S1, respectively. The league table showing the comparative efficacies shown as the risk ratio [RR] along with a 95% confidence interval [CI] is provided in Figure 4.

#### 3.4.2. Adverse Effects

**Figure 5:**
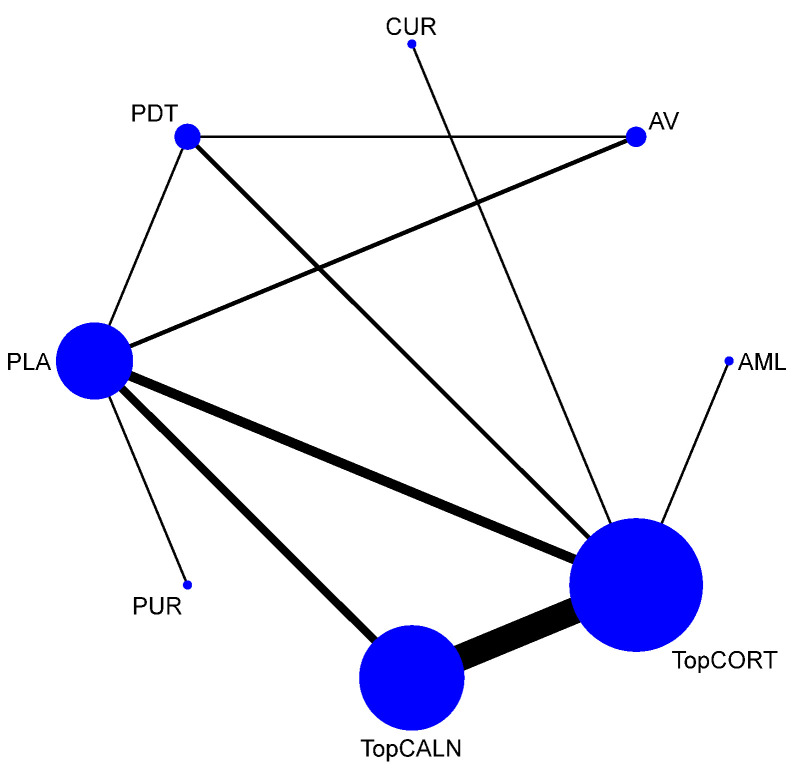
Network plot: Clinical Improvement of Oral Lichen Planus. Abbreviations: AML, amlexanox paste; AV, aloe vera; CUR, curcumin gel; PDT, photodynamic therapy; PLA, placebo; PUR, purslane; SysCORT, systemic corticosteroid; TopCALN, topical calcineurin; TopCALNcoSysCORT, topical calcineurin combined with systemic corticosteroid; TopCORT, topical corticosteroid.

**Figure 6:**
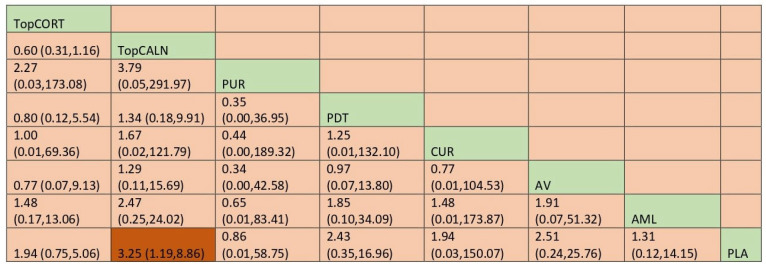
League table for adverse effects. Abbreviations: AML, amlexanox paste; AV, aloe vera; CUR, curcumin gel; PDT, photodynamic therapy; PLA, placebo; PUR, purslane; SysCORT, systemic corticosteroid; TopCALN, topical calcineurin; TopCALNcoSysCORT, topical calcineurin combined with systemic corticosteroid; TopCORT, topical corticosteroid. The green gihlights indicate the interventions and the dark highlights indicate the significant results.

In the case of adverse effects, there are 20 RCTs that reported those data. The network plot is provided in Figure 5. Network meta-analysis suggested that there was a significant adverse effect compared with placebo only for topical calcineurin [RR, 3.25 [95% CI: 1.19, 8.86]. When ranked, purslane was the best [SUCRA 63] and topical calcineurin was the worst [SUCRA 26.1], indicating the probability of the most adverse effects. Detailed results for SUCRA ranks and curves are presented in Supplementary Table S4 and Supplementary Figure S2, respectively. The league table showing the comparative safety shown as the risk ratio [RR] along with a 95% confidence interval [CI] is provided in Figure 6.

#### 3.4.3. SUCRA Value for Safety and Efficacy

**Figure 7:**
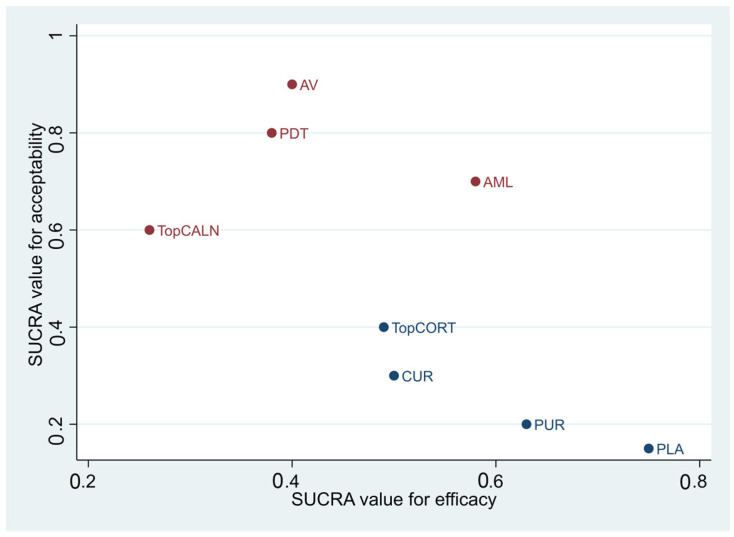
SUCRA Value for Safety and Efficacy of Interventions Used for Treatment of Oral Lichen Planus. Abbreviations: AV, aloe vera; PDT, photodynamic therapy; PUR, purslane; TopCALN, topical calcineurin; TopCORT, topical corticosteroid.

Figure 7 shows an overall analysis of the safety and efficacy of the interventions that were statistically significant. PUR was the safest and the most efficacious in treatment of OLP. TopCALN caused statistically significant adverse effects, therefore, it is ranked the lowest in safety. TopCORT was considered safe and effective in the treatment of OLP. 

#### 3.4.4. Clinical Resolution

**Figure 8:**
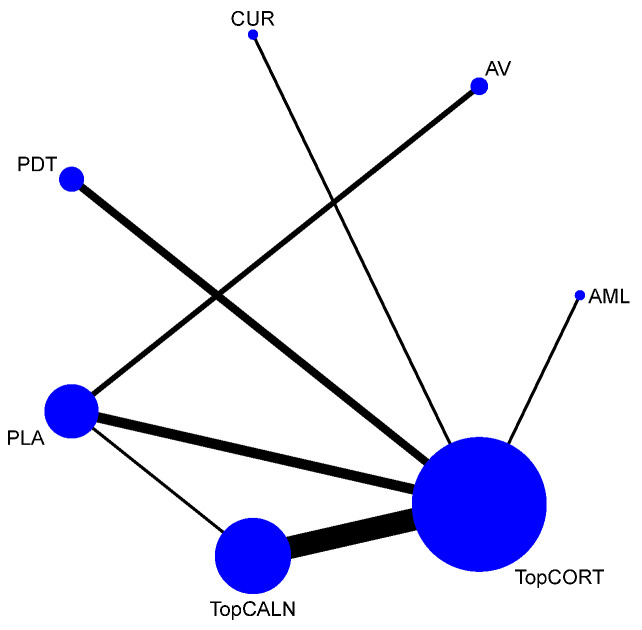
Network Plot: Clinical Resolution of Oral Lichen Planus. Abbreviations: AML, amlexanox paste; AV, aloe vera; CUR, curcumin gel; PDT, photodynamic therapy; PLA, placebo; TopCALN, topical calcineurin; TopCORT, topical corticosteroid.

**Figure 9:**
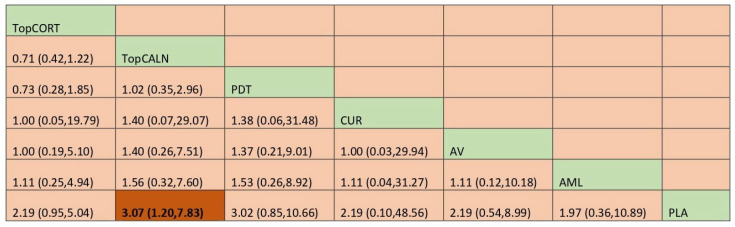
League table for adverse effects. Abbreviations: AML, amlexanox paste; AV, aloe vera; CUR, curcumin gel; PDT, photodynamic therapy; PLA, placebo; TopCALN, topical calcineurin; TopCORT, topical corticosteroid. The green highlights indicate the interventions and the dark highlights indicate the significant results.

Clinical resolution of OLP was considered as only when the lesion was completely healed. The network plot is provided in Figure 8. Network meta-analysis suggested that only TopCALN [RR = 3.07; 95% CI: 1.20, 7.83] was statistically significant in showing clinical resolution of OLP compared to placebo. Accordingly, on SUCRA ranking, TopCALN was ranked highest [SUCRA 72] for clinical resolution of OLP and TopCORT was the lowest [SUCRA 47.7] compared to placebo. Detailed results of SUCRA ranks and curves are presented in Supplementary Table S5 and Supplementary Figure S3. The league table showing the comparative efficacies shown as the risk ratio [RR] along with a 95% confidence interval [CI] is provided in Figure 9.

#### 3.4.5. Clinical Score

**Figure 10:**
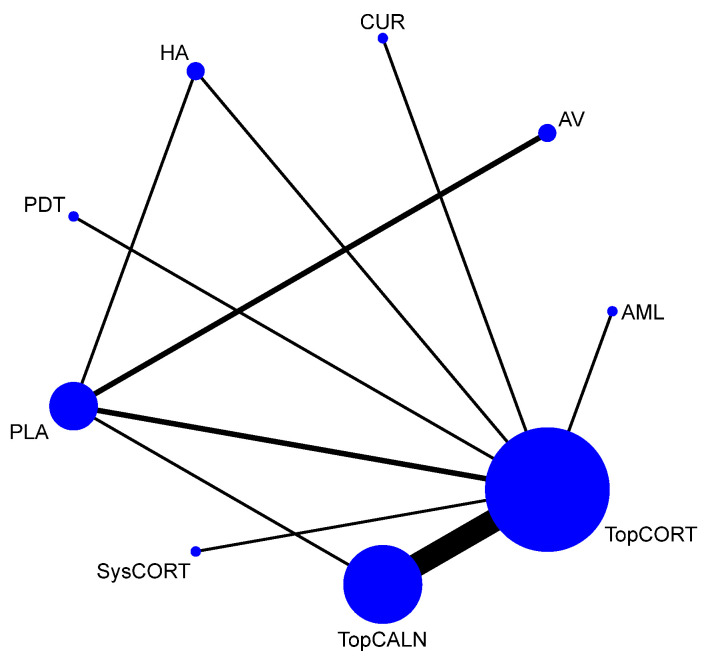
Network Plot: Clinical Score. Abbreviations: AML, amlexanox paste; AV, aloe vera; CUR, curcumin gel; PDT, photodynamic therapy; PLA, placebo; PUR, purslane; SysCORT, systemic corticosteroid; TopCALN, topical calcineurin; TopCORT, topical corticosteroid; HA, hyaluronic acid.

**Figure 11:**
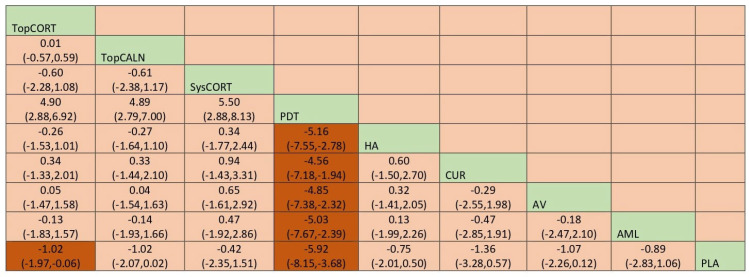
League table for clinical score. Abbreviations: AML, amlexanox paste; AV, aloe vera; CUR, curcumin gel; PDT, photodynamic therapy; PLA, placebo; PUR, purslane; SysCORT, systemic corticosteroid; TopCALN, topical calcineurin; TopCORT, topical corticosteroid; HA, hyaluronic acid. The green highlights indicate the interventions and the dark highlights indicate the significant results.

Improvement of clinical score for OLP was measured based on Thongprasom scale scoring. It is a scoring system for OLP based on the surface area involved and the severity of the lesion. The output results for clinical score were calculated using mean difference, and the network plot is presented in Figure 10. Based on the NMA findings, PDT [MD = −5.91 [95% CI: −8.15, –3.68] and TopCORT [MD = −1.02 [95% CI: −1.98, −0.06] showed statistically significant improvement in clinical score for OLP. When ranked, PDT demonstrated the highest score in improvement of clinical score for OLP [SUCRA 0] and SysCORT was ranked last [SUCRA 70.8]. Detailed SUCRA ranks and curves are presented in Supplementary Table S6 and Supplementary Figure S4. The league table showing the comparative efficacies shown as the risk ratio [RR] along with a 95% confidence interval [CI] is provided in Figure 11.

#### 3.4.6. Pain Score

**Figure 12:**
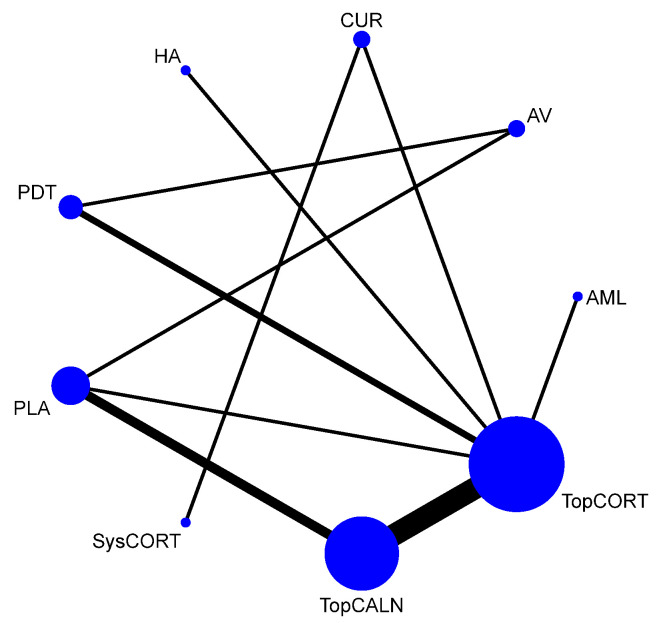
Network Plot: Pain Score. Abbreviations: AML, amlexanox paste; AV, aloe vera; CUR, curcumin gel; PDT, photodynamic therapy; PLA, placebo; PUR, purslane; SysCORT, systemic corticosteroid; TopCALN, topical calcineurin; TopCORT, topical corticosteroid; HA, hyaluronic acid.

**Figure 13:**
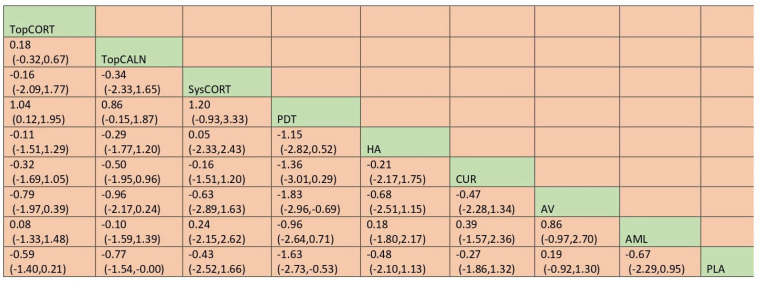
League table for pain score. Abbreviations: AML, amlexanox paste; AV, aloe vera; CUR, curcumin gel; PDT, photodynamic therapy; PLA, placebo; PUR, purslane; SysCORT, systemic corticosteroid; TopCALN, topical calcineurin; TopCORT, topical corticosteroid; HA, hyaluronic acid. The green highlights indicate the interventions and the dark highlights indicate the significant results.

Pain score was measured with visual analog scale [VAS] scoring. Based on the NMA (Figure 12), PDT showed statistically significant improvement in the pain score [MD −1.63, 95%CI: −2.73, −0.53]. In SUCRA ranks and curves, PDT was ranked the best in improving pain score [SUCRA 94.0], and AV ranked the worst [SUCRA 23.2]. Detailed SUCRA ranks and curves are presented in Supplementary Table S7 and Supplementary Figure S5. The league table showing the comparative efficacies shown as the mean deviation [MD] along with a 95% confidence interval [CI] is provided in Figure 13.

### 3.5. Subgroup Analysis of Individual Agents

#### 3.5.1. Clinical Improvement

**Figure 14:**
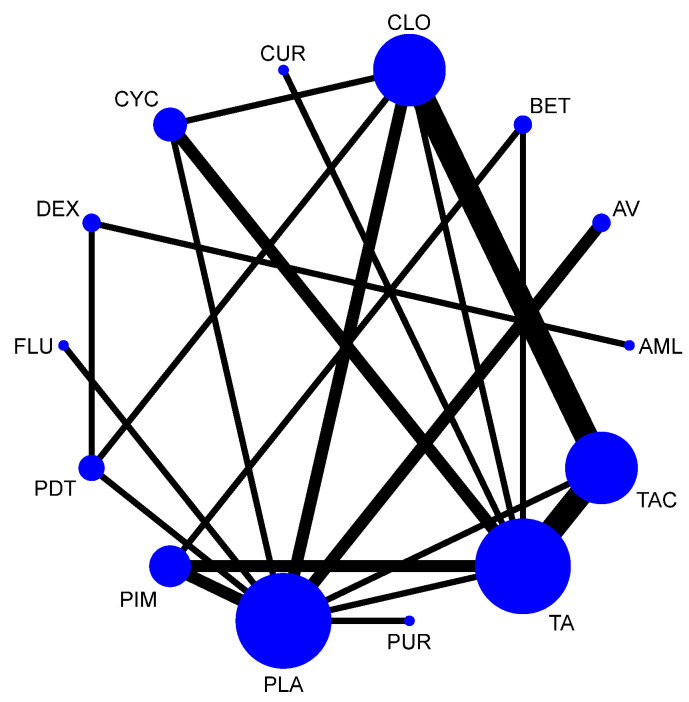
Network plot: Clinical Improvement (subgroup analysis). Abbreviations: AML, amlexanox paste; AV, aloe vera; CUR, curcumin gel; PDT, photodynamic therapy; PLA, placebo; PUR, purslane; TAC, tacrolimus; FLU, flucinonide acetonide; CLO, clobetasol propionate; PIM, pimecrolimus; BET, betamethasone; TA, triamcinolone acetonide; DEX, dexamethasone; CYC, cyclosporine.

**Figure 15:**
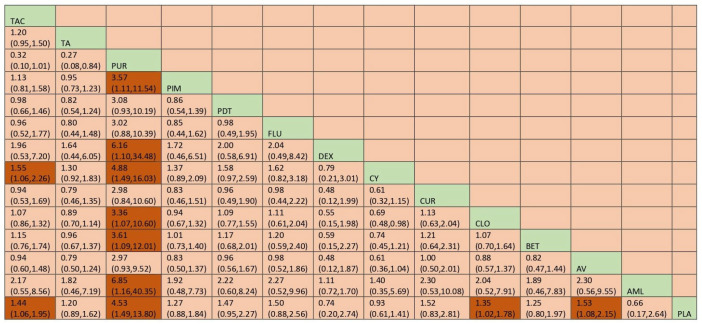
League table for clinical Improvement (subgroup analysis). Abbreviations: AML, amlexanox paste; AV, aloe vera; CUR, curcumin gel; PDT, photodynamic therapy; PLA, placebo; PUR, purslane; TAC, tacrolimus; FLU, flucinonide acetonide; CLO, clobetasol propionate; PIM, pimecrolimus; BET, betamethasone; TA, triamcinolone acetonide; DEX, dexamethasone; CYC, cyclosporine. The green highlights indicate the interventions and the dark highlights indicate the significant results.

The network plot for this subgroup analysis with individual agents for clinical outcome is presented in Figure 14. Network meta-analysis suggested that PUR [RR = 4.53; 95% CI: 1.49, 13.79], AV [RR = 1.53, 95% CI: 1.08, 2.15], TAC [RR = 1.43; 95% CI: 1.06, 1.95], and CLO [RR = 1.34; 95% CI: 1.02, 1.78] are statistically significant in clinical improvement of OLP. According to SUCRA ranking, PUR was ranked the first [SUCRA 98.1], followed by AV [SUCRA 68.5] and TAC [SUCRA 64.7]. Detailed SUCRA ranks of individual interventions and SUCRA curves are presented in Supplementary Table S8 and Supplementary Figure S6, respectively. The league table showing the comparative efficacies shown as the risk ratio [RR] along with a 95% confidence interval [CI] is provided in Figure 15.

#### 3.5.2. Adverse Effects

**Figure 16:**
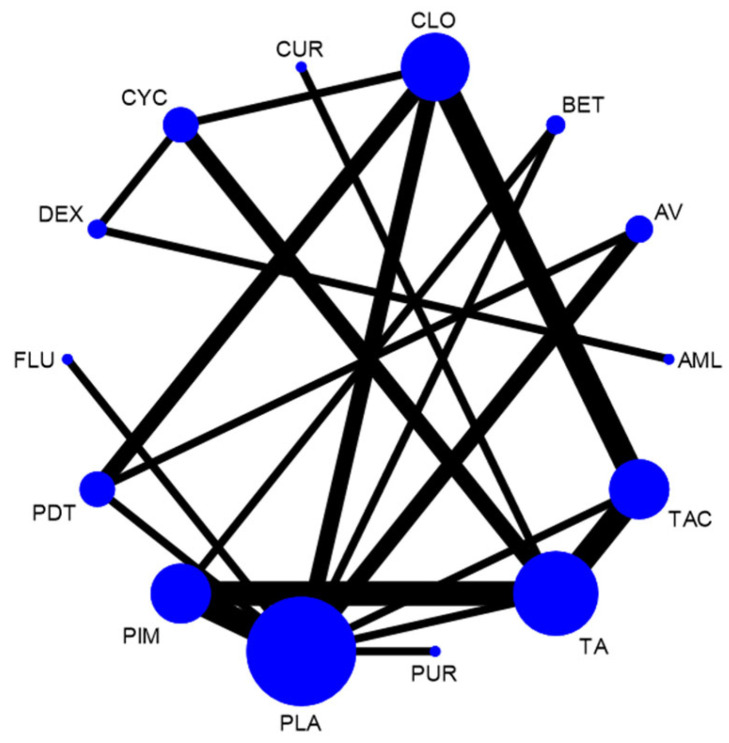
Network plot: Adverse Effects (subgroup analysis). Abbreviations: AML, amlexanox paste; AV, aloe vera; CUR, curcumin gel; PDT, photodynamic therapy; PLA, placebo; PUR, purslane; TAC, tacrolimus; FLU, flucinonide acetonide; CLO, clobetasol propionate; PIM, pimecrolimus; BET, betamethasone; TA, triamcinolone acetonide; DEX, dexamethasone; CYC, cyclosporine.

**Figure 17:**
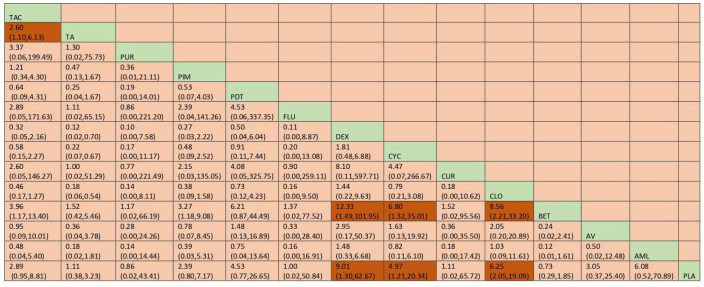
League table for adverse effects (subgroup analysis). Abbreviations: AML, amlexanox paste; AV, aloe vera; CUR, curcumin gel; PDT, photodynamic therapy; PLA, placebo; PUR, purslane; TAC, tacrolimus; FLU, flucinonide acetonide; CLO, clobetasol propionate; PIM, pimecrolimus; BET, betamethasone; TA, triamcinolone acetonide; DEX, dexamethasone; CYC, cyclosporine. The green highlights indicate the interventions and the dark highlights indicate the significant results.

Network meta-analysis suggested that CYC [RR = 4.96; 95% CI: 1.21, 20.34], DEX [RR = 9.01; 95% CI: 1.29, 62.67] and CLO [RR = 6.25; 95% CI: 2.04, 19.09] were statistically significant for adverse effects. According to SUCRA ranking, TA is the one with the least adverse effects, followed by CUR. Detailed SUCRA ranks of individual interventions and SUCRA curves are presented in Supplementary Table S9 and Supplementary Figure S7, respectively. The network plot is presented in Figure 16 and the league table showing the comparative efficacies is provided in Figure 17.

#### 3.5.3. Clinical Resolution

**Figure 18:**
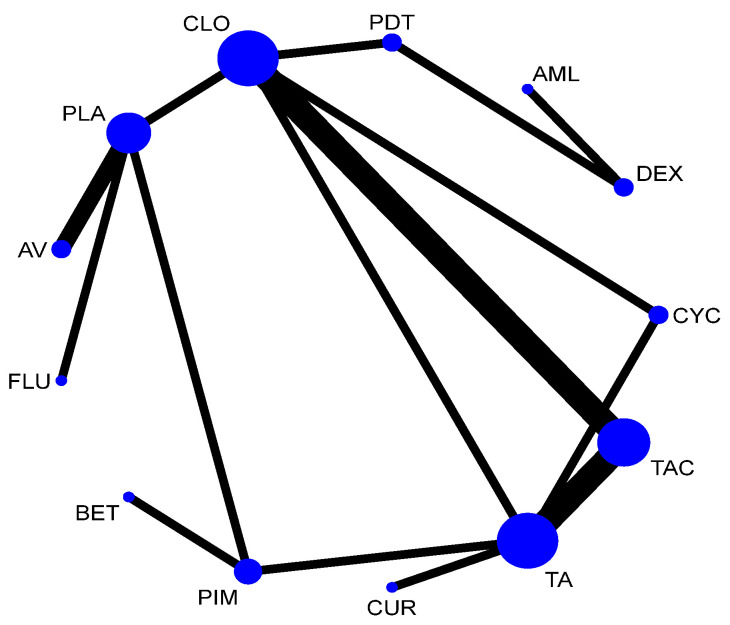
Network plot: Clinical Resolution (subgroup analysis). Abbreviations: AML, amlexanox paste; AV, aloe vera; CUR, curcumin gel; PDT, photodynamic therapy; PLA, placebo; PUR, purslane; TAC, tacrolimus; FLU, flucinonide acetonide; CLO, clobetasol propionate; PIM, pimecrolimus; BET, betamethasone; TA, triamcinolone acetonide; DEX, dexamethasone; CYC, cyclosporine.

**Figure 19:**
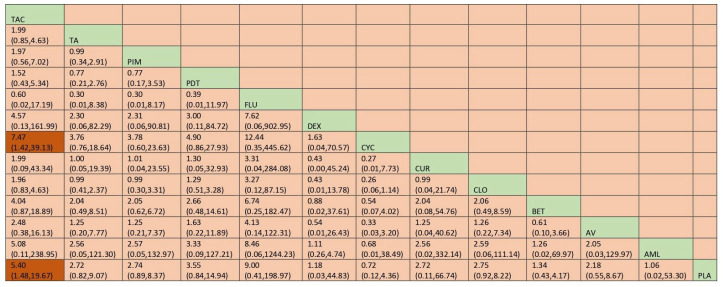
League table: Clinical Resolution (subgroup analysis). Abbreviations: AML, amlexanox paste; AV, aloe vera; CUR, curcumin gel; PDT, photodynamic therapy; PLA, placebo; PUR, purslane; TAC, tacrolimus; FLU, flucinonide acetonide; CLO, clobetasol propionate; PIM, pimecrolimus; BET, betamethasone; TA, triamcinolone acetonide; DEX, dexamethasone; CYC, cyclosporine. The green highlights indicate the interventions and the dark highlights indicate the significant results.

Based on the NMA findings, only TAC was significant in clinical resolution of OLP [RR = 5.40; 95% CI: 1.48, 19.67]. SUCRA ranks reported that TAC scored the highest [SUCRA 83], and CYC [SUCRA score: 17.8] scored the worst. Detailed SUCRA ranks of individual interventions and SUCRA curves are presented in Supplementary Table S10 and Supplementary Figure S8, respectively. The network plot is presented in Figure 18 and the league table showing the comparative efficacies is provided in Figure 19.

#### 3.5.4. Clinical Score

**Figure 20:**
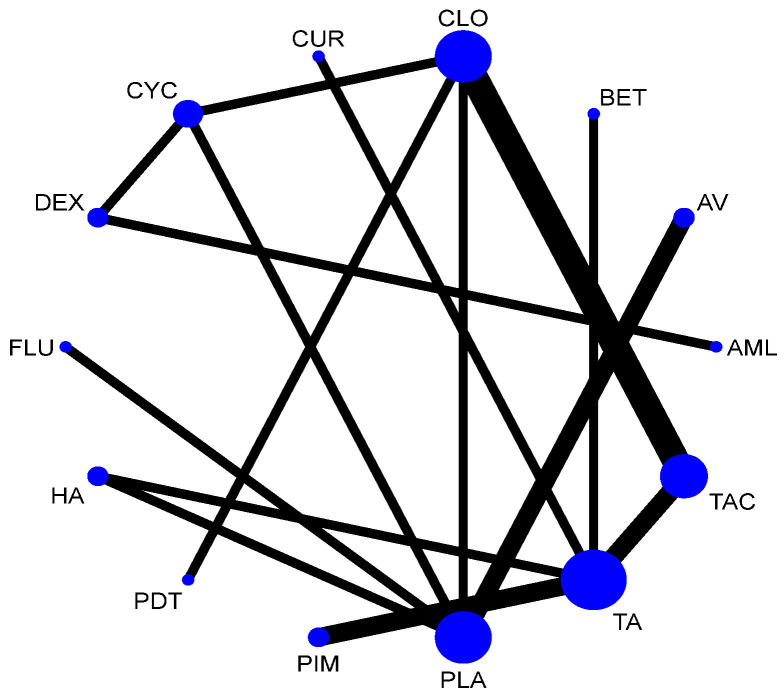
Network plot: Clinical Score (subgroup analysis). Abbreviations: AML, amelexanox paste; AV, aloe vera; CUR, curcumin gel; PDT, photodynamic therapy; PLA, placebo; PUR, purslane; TAC, tacrolimus; FLU, flucinonide acetonide; CLO, Clobetasol propionate; PIM, pimecrolimus; BET, betamethasone; TA, triamcinolone acetonide; DEX, dexamethasone; CYC, cyclosporine.

**Figure 21:**
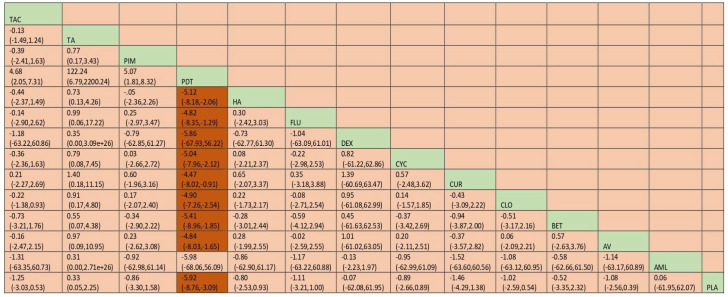
Network plot: Clinical Score (subgroup analysis). Abbreviations: AML, amelexanox paste; AV, aloe vera; CUR, curcumin gel; PDT, photodynamic therapy; PLA, placebo; PUR, purslane; TAC, tacrolimus; FLU, flucinonide acetonide; CLO, Clobetasol propionate; PIM, pimecrolimus; BET, betamethasone; TA, triamcinolone acetonide; DEX, dexamethasone; CYC, cyclosporine. The green highlights indicate the interventions and the dark highlights indicate the significant results.

None of the interventions were statistically significant in improving clinical score except PDT MD = −5.92; 95% CI: −8.76, 3.09]. When ranked, PDT scored the highest and BET scored the worst. Detailed SUCRA ranks of individual interventions and SUCRA curves are presented in Supplementary Table S11 and Supplementary Figure S9, respectively. The network plot is presented in Figure 20 and the league table showing the comparative efficacies with a 95% confidence interval [CI] is provided in Figure 21.

#### 3.5.5. Pain Score 

**Figure 22:**
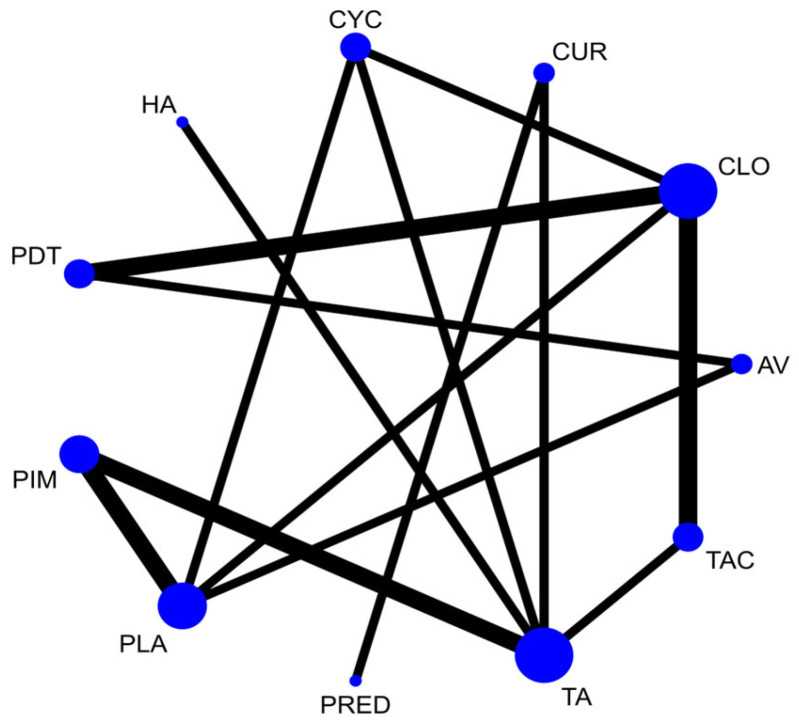
Network plot: Pain Score (subgroup analysis). Abbreviations: AML, amlexanox paste; AV, aloe vera; CUR, curcumin gel; PDT, photodynamic therapy; PLA, placebo; TAC, tacrolimus; FLU, flucinonide acetonide; CLO, clobetasol propionate; PIM, pimecrolimus; TA, triamcinolone acetonide; CYC, cyclosporine; PRED, prednisolone; HA, hyaluronic acid.

**Figure 23:**
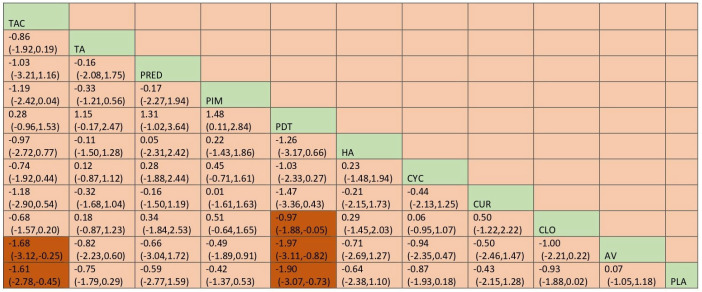
League table: Pain Score (subgroup analysis). Abbreviations: AML, amlexanox paste; AV, aloe vera; CUR, curcumin gel; PDT, photodynamic therapy; PLA, placebo; TAC, tacrolimus; FLU, flucinonide acetonide; CLO, clobetasol propionate; PIM, pimecrolimus; TA, triamcinolone acetonide; CYC, cyclosporine; PRED, prednisolone; HA, hyaluronic acid. The green highlights indicate the interventions and the dark highlights indicate the significant results.

Network meta-analysis suggested that TAC [MD = −1.67; 95% CI: −2.78, −48, SUCRA 86.3] and PDT [MD = −1.90 [95% CI: −3.06, −0.76, SUCRA 92.1] are statistically significant compared to placebo in improving the pain score. Detailed SUCRA ranks of individual interventions and curves are presented in Supplementary Table S12 and Supplementary Figure S10, respectively. The network plot is presented in Figure 22. The league table showing the comparative efficacies with a 95% confidence interval [CI] is provided in Figure 23.

### 3.6. Network Consistency and Small Study Effects and GRADE Quality

For all outcomes, the test of global inconsistency showed no evidence of inconsistency within any network comparisons. We did not find any evidence of publication bias on any outcome assessed based on the comparison-adjusted funnel plots, as all plots were symmetrical (Supplementary Figures S11–S20). The GRADE analysis of quality of evidence is provided in Supplementary Table S13. The evidence generated was classified as moderate quality evidence for all outcomes.

## 4. Discussion

Defining the most appropriate intervention used for treating OLP is challenging due to the wide variety of interventions that has been used to treat OLP, giving a wide range of beneficial results in various aspects. The majority of previous systematic reviews only conducted a pairwise comparison of interventions in clinical trials. The Cochrane Database of Systematic Reviews have also reported two reviews [12,13]. However, there is a lack of comprehensive evidence that compares the relative efficacy and safety of all interventions combined [1,11,25,30,31,32,42,49,50,51,52,53,54,55,56,58,59,60,61,62,63,64,65,66,67,68,69,70]. NMA enables the combination of both direct and indirect evidence to establish comparative efficacy and acceptability across a network of randomized controlled trials of all compounds [15]. A recent NMA has investigated the efficacy of topical administration for treatment of OLP [14]. The scope of the aforementioned review was restricted to topical agents only, whereas our review comprehensively evaluates all the available agents for the management of OLP. Further, our review has also included additional trials published in the recent couple of years. Therefore, our NMA presents the most recent cumulative evidence of comparative effectiveness of agents on symptomatic, biopsy-proven OLP on five major outcomes including clinical improvement, clinical resolution, adverse effects, clinical score and pain score. Further, the RCTs included in the present study included participants with clinically- and histologically-proven symptomatic forms of OLP, thus improving the reliability of the results. According to our results, purslane is the most effective agent, followed by aloe vera and topical calcineurin inhibitors in bringing clinical improvement. Topical calcineurin inhibitors are the best agents in terms of clinical resolution, however, they were the least safe. Photodynamic therapy was the most effective agent in terms of reduction in clinical score and pain score. 

Purslane, a type of green leafy vegetable in which plant extract was granulated with lactose and other inert substances, has the highest SUCRA ranking for clinical improvement of OLP and has the least adverse effects according to our analysis. However, this information was obtained from one study that was identified to have a high risk of bias [49]. It is prescribed to the patients in capsule form. The antioxidant activity of purslane is well established. This helps mitigate the oxidative stress brought about by inflammatory-cell-associated free radicals and reactive oxygen species [71,72,73]. Imbalance in antioxidant activity is reported in vulvar and skin LP [71,72]. Keratinocytes can also release ROS subsequent to stimulation by pro-inflammatory cytokines and endotoxins as well [71]. Both of the aforementioned can be reduced by the antioxidant activity of purslane. However, since the antioxidant activity of purslane has been demonstrated by only one study with a high risk of bias, more trials are required before a recommendation for routine use may be made. 

Aloe vera (AV) has been previously reported with only mild side effects restricted to mild itching and stinging sensation [50]. OLP is a T-cell-mediated disease and an autoimmune disease [51]. OLP upregulates adhesion through activated keratinocytes and lymphocytes which release interleukin [IL]-2, IL-4, IL-10 and tumour necrosis factor [TNF-alpha] [14,73,74]. AV acts by interfering with the arachidonic acid pathway via cyclooxygenase and by reducing TNF-alpha levels and leukocyte adhesion, thus contributing to the treatment of OLP [75,76,77,78]. However, more high-quality trials are recommended to be performed on AV for evidence synthesis, as there were only two trials with unclear risk of bias which were included in this review [50,51].

Calcineurin inhibitors impair transcription of interleukin [IL]-2 and several other cytokines in T lymphocytes [79]. TopCALN was found to be statistically significant in clinical improvement and clinical resolution of OLP. Clinical resolution is measured by the complete resolution of the lesion intraorally. TopCALN is usually recommended as the second line of therapy after the failure of TopCORT [10,55,80,81]. However, it has the highest incidence of adverse effects. The most common adverse effects caused by calcineurin inhibitors are transient burning or stinging sensation at the site of application [10,11,25,31,33,55,56,82]. Others include dysgeusia [82], mucosal paraesthesia [57] and gastrointestinal upsets [10,32]. However, a definitive conclusion on the long-term resolution cannot be made at this stage as most of the trials have a short follow-up period [10]. Moreover, systemic use of calcineurin has been linked to malignancy due to promotion of metastasis, tumour growth and angiogenesis [83]. A better understanding of adverse effects caused by calcineurin inhibitors is imperative before a recommendation for routine use may be made [10].

Corticosteroids have remained the treatment of choice for symptomatic OLP in many studies that had been conducted previously [34,84,85,86]. After balancing out all the outcomes from the results of the current study, our study also confirms that TopCORT is the most effective treatment option for OLP. It is statistically significant in causing clinical improvement of OLP and also in improving the clinical score. Clinical score is measured with Thongprasom scale scoring. Moreover, TopCORT is associated with adverse effects with lesser impact, including slight burning sensation [33,35,43,67], gastrointestinal upsets [43], xerostomia [44], candidiasis [58,65] and general discomfort [69]. This finding is in accordance with previous research which implicates corticosteroids as the most successful and predictable interventions in the treatment of OLP with minimal potential for systemic side effects [32,85,87,88,89,90]. Anti-inflammatory and immunosuppressive actions of corticosteroids contribute to its efficacy in the treatment of OLP [34,85,87]. Therefore, continued use of TopCORT for the treatment of OLP is recommended since it is implemented in current practice. 

After considering TopCORT, TopCALN, PUR and AV as clinically significant interventions that can be used for effective treatment of OLP, a subgroup analysis was performed for a more specific understanding of the interventions for treatment of OLP. TopCORT evaluated in this review were BET, CLO, DEX, FLU and TA. For TopCALN, PIM, CYC and TAC were included. It is observed that PUR, AV and TAC are statistically significant in the clinical improvement of OLP. They are also ranked top three in the SUCRA ranking. Based on the adverse effects, CLO and CYC can be seen being statistically significant in adverse effects. 

The current review has several strengths. In this review, different interventions were compared as a group as well as individually with a range of outcome measures. Efficacy and safety of a large number of interventions have been included in this review of both topical and systemic agents. Another strength of this NMA is that the comparison-adjusted funnel plot for clinical improvements of OLP and adverse effects were symmetrical, suggesting that results are not influenced by the sample size of literature and publication bias. 

Our study is not without limitations. A limitation of the current NMA is that it remains unclear in which concentration or dosage regimen represents “standard of care” for the recommended intervention. Dosages reported in individual trials varied. Outcomes were reported in different measurements when comparing the effectiveness and safety of interventions, which makes pooling the data challenging, Further the timing of the outcome measurements and of follow-up length varied between the studies. For meaningful comparison of data, there is a need to standardise parameters for the outcome measurement and the length of treatment OLP for intervention trials. International associations can keep standardised guidelines for the conduct of trials. In addition, future trials should focus on long-term follow up, particularly in terms of relapses. Cost-effectiveness and economic utility could also be considered as an area of research in in future trials to improve the clinical usefulness.

## 5. Conclusions

After performing the NMA with 37 studies, the available evidence suggests that purslane and aloe vera are the most effective drugs in the management for OLP, but only a small number of studies were performed on these interventions. There is also evidence that TopCALN is effective in treatment of OLP; however, the safety is a concern. The newer PDT is shown to be efficacious for pain and reduction in clinical scores and safe as well. Nevertheless, further RCTs are warranted to confirm and improve the accuracy of the findings from the present review.

## Data Availability

Supporting data is provided in the Supplementary Materials.

## Supplementary Materials

The following supporting information can be downloaded at: https://www.mdpi.com/article/10.3390/jcm12082763/s1. Table S1: Articles excluded and reasons for exclusion. Table S2: Characteristics of included RCTs.
