# Supplementary material for: Comparative Efficacy and Safety of Interventions for the Treatment of Oral Lichen Planus: A Systematic Review and Network Meta-Analysis
Source: J Clin Med. 2023 Apr 7;12(8):2763. doi: 10.3390/jcm12082763 (PMC10144824; doi:10.3390/jcm12082763)
Supplement: Supplementary file 1 [file jcm-12-02763-s001.zip › jcm-2236993-supplementary.pdf]

Supplementary Table S1. Articles excluded and reasons for exclusion

Study	Reason for Exclusion
Tyldesley 1977	Not all participants in study underwent biopsy
Rodstrom 1994	No quantitative data found that is suitable for analysis
Gaeta 1994	Article could not be found
Sieg 1995	No quantitative data found that is suitable for analysis
Xu 2002	Article was in Chinese Language
Hegarty 2002	No quantitative data found that is suitable for analysis
Wei 2003	Article could not be found
Campisi 2004	No quantitative data found that is suitable for analysis
Lodi 2007	Not a randomized clinical trial
Chainani-Wu 2007	No quantitative data found that is suitable for analysis
Carbone 2008	No quantitative data found that is suitable for analysis
Radfar 2008	Data is out of observation period of 4-6 weeks, and study included asymptomatic patients
Malhotra 2008	Adverse effects removed as number of patients with each adverse effect was not written specifically
Ghabanchi 2009	Multiple diseases included in the study
Mousavi 2009	Data was not clearly reported in graph
Xiong 2009	No quantitative data found that is suitable for analysis
Georgaki 2010	Article could not be found
McCaughey 2011	no quantitative data found that is suitable for analysis and asymptomatic patients included in study
Ahadian 2012	Data was not adequate for quantitative analysis
Luo 2013	Article could not be found
Sun 2013	Article could not be found
Zuo 2013	Article was in Chinese Language
Liu C 2013	No quantitative data found that is suitable for analysis

Amanat 2014	No quantitative data found that is suitable for analysis
Arduino 2014	No quantitative data found that is suitable for analysis
Pakferat 2015	Reporting VAS and clinical score values did not allow for quantitative analysis:
Azizi 2016	Not a randomized clinical trial (quasi-randomised)
Amirchaghmaghi 2016	Study included patients with no symptoms
Vohra 2016	No quantitative data found that is suitable for analysis
Mostafa 2017	Study included patients with no symptoms
Siponen 2017	Reporting VAS and clinical score values did not allow for quantitative analysis:
Hesen 2017	No quantitative data found that is suitable for analysis
Kanjanabuch 2017	No quantitative data found that is suitable for analysis
Thomas 2017	No quantitative data found that is suitable for analysis
Riaz 2017	Clincially diagnosed OLP & article could not be found
Singh 2017	No histological diagnosis for 16/40 patients
Ezzat 2019	Reporting VAS and clinical score values did not allow for quantitative analysis:
Villa 2020	No histological diagnosis for patients, only patients with VAS score 7 and above were included in study
Qataya 2020	No quantitative data found that is suitable for analysis
Samiee 2020	No quantitative data found that is suitable for analysis
Bernnardo 2020	Split mouth technique used
Santonocito (2020)	VAS score and clinical score did not allow for quantitative analysis
Z D Zhu 2021	Article was in Chinese Language
Rogulj 2021	Multiple diseases included in the study
Zborowski 2021	Split mouth technique used
Saglam 2021	No quantitative data found that is suitable for analysis
Agha-Hosseini 2021	no quantitative data found that is suitable for analysis
Hijazi 2021	Data was not clearly reported in graphs
Ferri (2021)	Clinical score did not allow for quantitiative analysis

Saengprasittchok 2022	Primary and secondary outcomes did not fulfill our criteria
Mamadapur 2022	No histological diagnosis for patients

Supplementary Table S2. Characteristics of included RCTs

Author (Year)	Country	Study Design	Diagnosed by	Type of OLP	Sample Size after Exclusion	Study Comparison	Duration of Treatment	Administration Method	Time of evaluations and assessments	Outcome: Clinical Improvement	Outcome: Clinical Resolution	Outcome: Adverse Effects	Outcome: Pain Score	Outcome: Clinical Score
Hashem (2019)	Egypt	randomized controlled trial	confirmed by histopathology, symptomatic OLP	erosive, atrophic, combined	40	TA vs HA	28 days	topical	1. pre-treatment 2.14 days 3. 21 days 4. 28 days	nil	nil	nil	mean: 1.3 ; 1.4 sd: 0.9 ; 0.9	mean: 0.5 ; 0.6 sd: 0.4 ; 0.5
Ezzat (2019)	Egypt	prospective, randomized, double blind, parallel design clinical trial (RCT)	clinical and histological diagnosis of OLP	erosive or atrophic	30	PIM vs BET	4 weeks	topical	1. pre-treatment 2. 1 week 3. 2 weeks 4. 4 weeks 5. after 4 weeks of treatment free period	15/15 ; 15/15	9/15 ; 4/15	6/15 ; 3/15	nil	nil

Arduino (2018)	Italy	randomized, placebo-controlled, double blind study	histological diagnosis based on WHO criteria	erosive	32	CLO vs PLA	8 weeks	topical	1. pre-treatment 2. After 4 weeks 3. After 8 weeks 4. After 24 weeks	14/16 ; 10/16	1/16 ; 0/16	2/16 ; 1/16	mean: 1.4 ; 3.2 sd: 1.7 ; 3.1	mean: 2.7 ; 3.1 sd: 0.9 ; 0.6
Bakhtiari (2017)	Iran	randomized clinical trial (RCT)	clinical and histological diagnosis of OLP	reticular or erosive	30	PDT vs DEX	2 weeks	topical	1. pre-treatment 2. 15 days 3. 30 days 4. 60 days 5. 90 days	6/15 ; 3/15	1/15 ; 0/15	nil	nil	nil
Hettiarachchi (2017)	Sri Lanka	randomized, comparative, double blind clinical trial study (RCT)	histolgically confirmed symptomatic OLP	reticular or erosive	68	TAC vs CLO	3 weeks	topical	1. pre-treatment 2. 3 weeks after treatment commencement 3. 5 weeks after treatment commencement	11/34 ; 9/34	nil	0/34 ; 0/34	mean: 0.7 ; 0.8 sd: 0.8 ; 1	mean: 1.9 ; 1.8 sd: 0.9 ; 1
Siponen (2017)	Finland	randomized, multicentre, double blind,	clinical and histological diagnosis of OLP	erosive	27	TAC vs TA vs PLA	3 weeks	topical	1. pre-treatment 2. week 3 3. week 6	11/11 ; 7/7 ; 9/9	nil	8/11 ; 3/7 ; 3/9	nil	nil

		placebo-controlled parallel randomized controlled trial							4. week 9<br>5. follow up visit at 6 months					
Sivaraman (2016)	India	randomized controlled trial (RCT)	clinical and histological diagnosis of OLP	reticular and erosive	30	CLO vs TA vs TAC	6 weeks	topical	1. pre-treatment<br>2. week 1<br>3. week 3<br>4. week 6<br>5. after 3 months	10/10 ; 10/10 ; 8/10	10/10 ; 10/10 ; 8/10	nil	nil	nil
Arunkumar (2015)	India	prospective, parallel group, randomized, active controlled clinical study (RCT)	clinical and histological diagnosis of OLP	erosive, ulcerative or symptomatic	30	TA vs PIM	2 months	topical	1. pre-treatment<br>2. 1st month<br>3. 2nd month<br>4. 4th month	15/15 ; 15/15	nil	0/15 ; 0/15	mean: 4 ; 4<br>sd: 4.3 ; 4.3	mean: 0.6 ; 0.9<br>sd: 0.5 ; 0.6
Kia (2015)	Iran	randomized clinical trial (RCT)	clinical and histological diagnosis of OLP	atrophic and ulcerative	50	CUR vs TA	4 weeks	topical	1. pre-treatment<br>2. at the end of 2 weeks<br>3. at the end of 4 weeks	19/25 ; 15/25	1/25 ; 1/25	0/25 ; 0/25	mean: 2.6 ; 1.8<br>sd: 3 ; 1.8	mean: 2.6 ; 3<br>sd: 1.3 ; 1

Pakfetrat (2015)	Itan	single blind, clinical trial	histopathologically confirmed OLP	atrophic or erosive	28	PIM vs TA	2 months	topical	1. pre-treatment 2. every 2 weeks for 2 months (5 visits)	12/14 ; 12/14	10/14 ; 12/14	nil	nil	nil
Dillenburg (2014)	Brazil	single-centre, randomized controlled, single-blind study (RCT)	symptomatic OLP and histological diagnosis of OLP based on WHO criteria	atrophic or erosive	42	PDT vs CLO	30 days	topical	1. pre-treatment 2. day 7 3. day 14 4. day 21 5. day 30 6. day 60 7. day 90	21/21 ; 21/21	13/21 ; 6/21	0/21 ; 5/21	mean: 1.1 ; 1.7 sd: 0.3 ; 0.4	mean: 1 ; 1.5 sd: 0.1 ; 0.1
Fu (2012)	West China	randomized, positive-controlled clinical trial (RCT)	clinical and histological diagnosis of OLP	erosive	38	AML vs DEX	7 days	topical	1. day 0 2. day 7	17/20 ; 17/18	7/20 ; 7/18	3/20 ; 4/18	mean: 1 ; 1.1 sd: 1.3 ; 1.3	mean: 6.1 ; 5.1 sd: 8.5 ; 6.3
Revanappa (2012)	India	randomized controlled study	histopathologically confirmed OLP, symptomatic OLP	erosive and atrophic	60	TAC vs TA	4 weeks	topical	1. pre-treatment 2. 14 days treatment 3. evaluation end of every week for 4 consecutive weeks	28/30 ; 16/30	20/30 ; 3/30	0/30 ; 0/30	mean: 1.2 ; 2.7 sd: 2.6 ; 2.5	mean: 2.3 ; 1.2 sd: 1.1 ; 1.4

Agha-Hossein i (2010)	Iran	random ized, double blind, placebo - controll ed trial (RCT)	clinical and histological diagnosis of OLP	erosive	37	PUR vs PLA	6 month s	topical	1. pre-treatment<br>2. 2 weeks<br>3. 1 month<br>4. 2 months<br>5. 3 months<br>6. 4 months<br>7. 5 months<br>8. 6 months	16/20 ; 3/17	nil	0/20 ; 0/17	nil	nil
Salazar-Sanchez (2010)	Spain	random ized, double blind study, placebo - controll ed trial (RCT)	clinical and histological diagnosis of OLP	sympto matic erosive or ulcerativ e	55	AV vs PLA	12 weeks	topical	1. pre-treatment<br>2. 6 weeks<br>3. 12 weeks	29/32 ; 20/29	10/32 ; 5/29	0/32 ; 0/29	mean: 2.7 ; 3.4 sd: 2.8 ; 3	mean: 1.7 ; 1.8 sd: 1.3 ; 1.2
Nolan (2009)	UK	random ized, double blind study, placebo - controll ed trial (RCT)	clinical and histological diagnosis of OLP	erosive or atrophic and ulcerativ e	113	HA vs PLA	28 days	topical	1. pre-treatment<br>2. 15 days<br>3. 29 days	nil	nil	nil	nil	mean: 13 ; 13.5 sd: 0.7 ; 0.7

Choonhakarn (2008)	Thailand	randomized, double blind study, placebo-controlled trial (RCT)	clinical and histological diagnosis of OLP	atrophic and erosive	54	AV vs PLA	8 weeks	topical	1. pre-treatment 2. 2 weeks 3. 4 weeks 4. 6 weeks 5. 8 weeks	26/27 ; 14/27	2/27 ; 0/27	2/27 ; 0/27	nil	mean: 1.1 ; 2.4 sd: 0.6 ; 0.6
Corrocher (2008)	Italy	randomized, controlled, double blind clinical trial (RCT)	clinical and histological diagnosis of OLP, symptomatic OLP	moderate or severe	32	TAC vs CLO	4 weeks	topical	1. pre-treatment 2. 4 weeks after beginning treatment 3. 6 weeks after beginning treatment (2 weeks after discontinuing treatment)	16/16 ; 16/16	9/16 ; 0/16	9/16 ; 0/16	mean: 0.3 ; 1.3 sd: 0.5 ; 0.6	mean: 0.4 ; 1.1 sd: 0.5 ; 0.3
Malhotra (2008)	India	randomized clinical trial (RCT)	clinical and histological diagnosis of OLP	erosive	46	BET vs TA	6 months	systemic	1. pre-treatment 2. 2 weeks 3. 4 weeks 4. 8 weeks 5. 12	17/25 ; 16/24	nil	nil	nil	mean: 3.1 ; 2 sd: 1.9 ; 1.7

									weeks<br>6. 16 weeks<br>7. 20 weeks<br>8. 24 weeks					
Volz (2008)	Germany	prospective, double blind, randomized, placebo-controlled trial (RCT)	clinical and histological diagnosis of OLP	erosive	20	PIM vs PLA	30 days	topical	1. pre-treatment<br>2. after 30 days (end of blinded treatment period)<br>3. after 60 days (end of follow up or end of open label treatment period)	7/10 ; 4/10	7/10 ; 2/10	5/10 ; 1/10	nil	nil
Gourohi (2007)	Iran	investigator blinded parallel group, randomized clinical trial (RCT)	histologically confirmed OLP	reticular, erosive or ulcerative, erythematous or atrophic	35	PIM vs TA	2 months	topical	1. pre-treatment<br>2. Weekly visits<br>3. 2 months of treatment free observation	nil	nil	2/18 ; 0/17	mean: -8.8, -9.3 sd: 11.1 ; 18	mean: -0.7 ; -0.7 sd: 0.6 ; 0.7

Passeron (2007)	France	double blind, randomized, prospective trial with placebo control (RCT)	clinical and histological diagnosis of OLP	erosive	12	PIM vs PLA	4 weeks	topical	1. Day 0<br>2. Day 14<br>3. Day 28	5/6 ; 3/6	nil	2/6 ; 0/6	mean: 0.8 ; 0.7 sd: 1 ; 0.8	nil
Thongprasom (2007)	Thailand	randomized controlled trial (RCT)	clinical and histological diagnosis of OLP, symptomatic OLP	atrophic-erosive, symptomatic	12	CYC vs TA	8 weeks	topical	1. pre-treatment<br>2. week 2<br>3. week 4<br>4. week 8<br>5. follow up after 12 weeks and every 3 months up till a year	2/6 ; 6/6	0/6 ; 3/6	6/6 ; 0/6	nil	nil
Conrotto (2006)	Italy	randomized, comparative, controlled, double blind study (RCT)	clinical and histological diagnosis of OLP	atrophic or erosive	39	CLO vs CYC	2 months	topical	1. pre-treatment<br>2. every 2 weeks during 2 month treatment and 2 month follow up	18/19 ; 13/20	9/19 ; 3/20	6/19 ; 1/20	mean: 1.8 ; 2.3 sd: 2.1 ; 1.7	mean: 2 ; 3.1 sd: 1.2 ; 1.6

Laeijendecker (2006)	Netherlands	prospective, randomized study (RCT)	clinical and histological diagnosis of OLP	erosive or ulcerative, atrophic or erythematous, hyperkeratotic	40	TAC vs TA	6 weeks	topical	1. pre-treatment 2. after 6 weeks	18/20 ; 9/20	6/20 ; 2/20	8/20 ; 3/20	nil	mean: 0.8 ; 0.6 sd: 1.5 ; 0.7
Yoke (2006)	Singapore, Korea, India, Thailand	randomized controlled trial (RCT)	clinical and histological diagnosis of OLP, symptomatic OLP	erosive, reticular, atrophic, mixed, combinations	139	CYC vs TA	8 weeks	topical	1. Week 0 2. Week 2 3. Week 4 4. Week 8	29/68 ; 41/71	nil	19/68 ; 3/71	mean: 1.6 ; 1.3 sd: 2.4 ; 2.3	nil
Swift (2005)	USA	randomized controlled trial (RCT)	histologically diagnosed, direct immunofluorescence	erosive	18	PIM vs PLA	4 weeks	topical	1. pre-treatment 2. midpoint (week 2) 3. final (week 4)	nil	nil	1/10 ; 0/10	mean: 2.1 ; 2.4 sd: 2.3 ; 1.6	nil
Lundquist (1995)	Sweden	comparative randomized study (RCT, split mouth)	clinical and histological diagnosis of OLP, symptomatic OLP	reticular, papular, plaque, bullous, atrophic, erosive	18	PDT vs PLA	24-36 days	topical	1. pre-treatment 2. end of treatment 3. 1 month follow up 4. 6 months follow up 5. 12 months	13/18 ; 6/18	nil	16/18 ; 0/18	nil	nil

									follow up					
Voute (1993)	Nether lands	random ized, double blind, placebo - controll ed trial (RCT)	clinical, histopatholo gic and immunofluo resence microscopy findings	erosive, reticular , atrophic , mixed, combina tions	40	FLU vs PLA	9 weeks	topical	1. pre-treatment 2. 3 weeks after 3. 6 weeks 4. 9 weeks	18/20 ; 12/20	4/20 ; 0/20	0/20 ; 0/20	nil	mean: 1.5 ; 2.8 sd: 1.2 ; 1.1
Eisen (1990)	USA	random ized, double blind analysi s (RCT)	histologicall y confirmed OLP	erosive/ erythem atous (sympto matic)	16	CYC vs PLA	8 weeks	topical	1. pre-treatment 2. biweekly thereafte r	8/8 ; 1/8	nil	3/20 ; 7/20	mean: 0.4 ; 1.6 sd: 0.6 ; 0.8	mean: 0.3 ; 1.7 sd: 0.3 ; 1.1
Sonthali a (2012)	India	random ized, double blind trial	histologicall y diagnosed OLP	erosive or ulcerativ e	40	TAC vs CLO	8 weeks	topical	1. pre-treatment 2. 2 weeks 3. 4 weeks 4. 8 weeks 5. post treatment at 12 weeks	19/20 ; 14/20	14/20 ; 8/20	0/14 ; 0/14	nil	mean: 3.2 ; 1.7 sd: 2.8 ; 1.3
Bhatt (2021)	India	random ized clinical trial	clinical & histological diagnosis	reticular , atrophic ,	60	AV vs PDT	2 month s	Topical	1. pre-treatment 2. 2 months	nil	nil	0/30 ; 0/30	mean: 4.5 ; 3.8 sd: 0.3 ; 0.3	nil

				erosive, papular					3. 9 months					
Samimi (2020)	France	randomized, double-blind, controlled study	clinically & histologically confirmed based on WHO criteria	erosive	76	PLA vs BET	3 months	Topical	1. pre-treatment 2. 3 months 3. 6 months	nil	9/33 ; 13/33	17/33 ; 10/33	nil	nil
Ferri (2020)	Brazil	randomized, controlled, double-blind study	clinical & histological diagnosis	atrophic, reticular, erosive	34	PDT vs CLO	30 days	Topical	1. pre-treatment 2. once a week during treament (for 30 days) 3. 30 days after treatment 4. 60 days after treatment 5. 90 days after treatment	nil	12/17 ; 14/17	0/17 ; 0/17	mean: 2.1 ; 1.9 sd: 2.0 ; 2.2	nil
Kia (2020)	Iran	double blinded randomized clinical trial	clinical & histological diagnosis	erosive & atrophic	60	CYR vs PRED	1 month	Systemic	1. pre-treatment 2. 1st week 3. 2nd week	nil	nil	nil	mean: 2.7 ; 2.3 sd: 2.9 ; 2.0	nil

									3. 4th week					
Santonocito (2020)	Italy	randomized controlled clinical trial	clinical & histological diagnosis	erosive & atrophic	40	CLO vs PLA	3 months	Topical	1. pre-treatment 2. after 3 months	16/20 ; 12/20	13/20 ; 6/20	6/20 ; 0/20	nil	nil
Georgaki (2021)	Greece	randomized controlled study	clinical & histological diagnosis	erosive & atrophic	32	DEX vs CYC	1 month	Topical	1. pre-treatment 2. every week for the first month 3. every 15 days for the second month 4. once a month for the subsequent 4 months	nil	nil	7/18 ; 3/14	mean: 1.3 ; 2.1 sd: nil	mean: 2.1 ; 2.6 sd: nil

Supplementary Table S3. Results of network meta-analysis: Clinical Improvement of Oral Lichen Planus with SUCRA ranking

**Interventions**	**Clinical Improvement in Oral Lichen Planus**		
	**Relative Risk (95% CI)**	**P-value**	**SUCRA rank (Score)**
**PUR**	4.53 (1.46– 14.11)	**0.010***	1 (96.5)
**CUR**	1.71 (0.91- 3.22)	0.095	2 (67.4)
**PDT**	1.58 (1.01- 2.46)	**0.044***	3 (62.1)
**AV**	1.53 (1.05- 2.24)	**0.028***	4 (57.2)
**TopCALN**	1.39 (1.06- 1.81)	**0.016***	5 (47.3)
**SysCORT**	1.38 (0.73- 2.59)	0.319	6 (43.0)
**TopCORT**	1.35 (1.06- 1.73)	**0.017***	7 (40.5)
**AML**	1.22 (0.71-2.09)	0.478	8 (30.0)
**PLA**	Reference		9 (6.0)

Note: presence of (*) and texts in red indicates intervention is statistically significant Abbreviations: AML, amlexanox paste; AV, aloe vera; CUR, curcumin gel; PDT, photodynamic therapy; PLA, placebo; PUR, purslane; SysCORT, systemic corticosteroid; TopCALN, topical calcineurin; TopCORT, topical corticosteroid

Supplementary Table S4. Results of network meta-analysis: Adverse Effects with SUCRA ranking.

**Interventions**	**Adverse Effects**		
	**Relative Risk (95% CI)**	**P-value**	**SUCRA rank (Score)**
**PLA**	Reference		1 (74.9)
**PUR**	0.86 (0.01- 58.75)	0.943	2 (63.0)
**AML**	1.31 (0.12- 14.15)	0.822	3 (58.4)
**CUR**	1.94 (0.03-150.07)	0.764	4 (50.3)
**TopCORT**	1.94 (0.75- 5.06)	0.173	5 (49.1)
**AV**	2.51 (0.24- 25.76)	0.438	6 (40.1)
**PDT**	2.43 (0.35- 16.96)	0.370	7 (38.1)
**TopCALN**	3.25 (1.19- 8.86)	**0.022***	8 (26.1)

Note: presence of (*) and texts in red indicates intervention is statistically significant. Abbreviations: AML, amlexanox paste; AV, aloe vera; CUR, curcumin gel; PDT, photodynamic therapy; PLA, placebo; PUR, purslane; SysCORT, systemic corticosteroid; TopCALN, topical calcineurin; TopCALNcoSysCORT, topical calcineurin combined with systemic corticosteroid; TopCORT, topical corticosteroid

Supplementary Table S5. Results of network meta-analysis: Clinical Resolution of Oral Lichen Planus with SUCRA ranking.

**Interventions**	**Clinical Resolution of Oral Lichen Planus**		
	**Relative Risk (95% CI)**	**P-value**	**SUCRA rank (Score)**
**TopCALN**	3.07 (1.20- 7.83)	**0.019***	1 (72.0)
**PDT**	3.02 (0.85- 10.66)	0.086	2 (69.0)
**AV**	2.19 (0.54-8.99)	0.274	3 (50.6)
**CUR**	2.19 (0.10- 48.56)	0.620	4 (50.0)
**AML**	1.97 (0.36- 10.89)	0.437	5 (48.8)
**TopCORT**	2.19 (0.95- 5.04)	0.066	6 (47.7)
**PLA**	Reference		7 (12.0)

Note: presence of (*) and texts in red indicates intervention is statistically significant. Abbreviations: AML, amlexanox paste; AV, aloe vera; CUR, curcumin gel; PDT, photodynamic therapy; PLA, placebo; TopCALN, topical calcineurin; TopCORT, topical corticosteroid.

Supplementary Table S6 : Results of network meta-analysis: Clinical Score with SUCRA ranking.

**Interventions**	**Clinical Score**		
	**Mean Difference (95% CI)**	**P-value**	**SUCRA rank (Score)**
**PLA**	Reference		1 (89.7)
**SysCORT**	-0.42 (-2.35- 1.51)	0.672	2 (70.8)
**HA**	-0.75 (-2.01- 0.50)	0.240	3 (57.2)
**AML**	-0.89 (-2.83- 1.06)	0.373	4 (55.4)
**TopCALN**	-1.02 (-2.07- 0.02)	0.055	5 (46.9)
**AV**	-1.07 (-2.26- 0.12)	0.078	6 (46.1)
**TopCORT**	-1.02 (-1.97- (-0.06))	**0.037***	7 (46.0)
**CUR**	-1.36 (-3.28- 0.57)	0.167	8 (37.8)
**PDT**	-5.92 (-8.15- (-3.68))	**0.000***	9 (0.0)

Note: presence of (*) and texts in red indicates intervention is statistically significant. Abbreviations: AML, amlexanox paste; AV, aloe vera; CUR, curcumin gel; PDT, photodynamic therapy; PLA, placebo; PUR, purslane; SysCORT, systemic corticosteroid; TopCALN, topical calcineurin; TopCORT, topical corticosteroid; HA, hyaluronic acid.

Supplementary Table S7. Results of network meta-analysis: Pain Score with SUCRA ranking.

**Interventions**	**Pain Score**		
	**Mean Difference (95% CI)**	**P-value**	**SUCRA rank (Score)**
**PDT**	-1.63 (-2.73- (-0.53))	**0.004***	1 (94.1)
**TopCALN**	-0.77 (-1.54- (-0.00)	**0.049***	2 (67.0)
**AML**	-0.67 (-2.29- 0.95)	0.420	3 (56.4)
**TopCORT**	-0.59 (-1.40- 0.21)	0.150	4 (55.4)
**HA**	-0.48 (-2.10-1.13)	0.557	5 (48.9)
**SysCORT**	-0.43 (-2.52- 1.66)	0.686	6 (48.1)
**CUR**	-0.27 (-1.86- 1.32)	0.736	7 (36.8)
**PLA**	Reference		8 (24.5)
**AV**	0.19 (-0.92- 1.30)	0.731	9 (18.8)

Note: presence of (*) and texts in red indicates intervention is statistically significant. Abbreviations: AML, amlexanox paste; AV, aloe vera; CUR, curcumin gel; PDT, photodynamic therapy; PLA, placebo; PUR, purslane; SysCORT, systemic corticosteroid; TopCALN, topical calcineurin; TopCORT, topical corticosteroid; HA, hyaluronic acid.

Supplementary Table S8. Results of network meta-analysis: Clinical improvement of Oral Lichen Planus (subgroup analysis) with SUCRA ranking.

**Interventions**	**Clinical Improvement in Oral Lichen Planus (Subgroup Analysis)**		
	**Relative Risk (95% CI)**	**P-value**	**SUCRA rank (Score)**
**PUR**	4.53 (1.49- 13.80)	**0.008***	1 (98.1)
**CUR**	1.52 (0.83- 2.81)	0.178	2 (68.6)
**AV**	1.53 (1.08- 2.15)	**0.016***	3 (68.5)
**PDT**	1.47 (0.95- 2.27)	0.082	4 (66.3)
**TAC**	1.44 (1.06- 1.95)	**0.019***	5 (64.7)
**FLU**	1.5 (0.88-2.56)	0.137	6 (64.6)
**CLO**	1.35 (1.02- 1.78)	**0.037***	7 (55.6)
**PIM**	1.27 (0.88- 1.84)	0.206	8 (48.3)
**BET**	1.25 (0.80- 1.97)	0.324	9 (44.8)
**TA**	1.20 (0.89- 1.62)	0.222	10 (39.4)
**DEX**	0.74 (0.20-2.74)	0.647	11 (24.8)
**PLA**	Reference		12 (20.1)
**AML**	0.66 (0.17- 2.64)	0.559	13 (19.1)
**CYC**	0.93 (0.61- 1.41)	0.728	14 (17.0)

Note: presence of (*) and texts in red indicates intervention is statistically significant. Abbreviations: AML, amlexanox paste; AV, aloe vera; CUR, curcumin gel; PDT, photodynamic therapy; PLA, placebo; PUR, purslane; TAC, tacrolimus; FLU, flucinonide acetonide; CLO, clobetasol propionate; PIM, pimecrolimus; BET, betamethasone; TA, triamcinolone acetonide; DEX, dexamethasone; CYC, cyclosporine.

Supplementary Table S9. Results of network meta-analysis: Adverse Effects (subgroup analysis) with SUCRA ranking.

**Interventions**	**Adverse Effects (Subgroup Analysis)**		
	**Relative Risk (95% CI)**	**P-value**	**SUCRA rank (Score)**
**BET**	0.73 (0.29- 1.85)	0.507	1 (84.3)
**PLA**	Reference		2 (76.9)
**TA**	1.11 (0.38- 3.23)	0.845	3 (74.0)
**CUR**	1.11 (0.02- 65.72)	0.959	4 (66.2)
**PUR**	0.86 (0.02- 43.41)	0.939	5 (65.6)
**FLU**	1.00 (0.02- 50.84)	1.000	6 (63.3)
**PIM**	2.39 (0.80- 7.17)	0.120	7 (51.0)
**TAC**	2.89 (0.95- 8.81)	0.062	8 (45.2)
**AV**	3.05 (0.37- 25.40)	0.301	9 (44.3)
**PDT**	4.53 (0.77- 26.65)	0.094	10 (32.4)
**CYC**	4.97 (1.21- 20.34)	**0.026***	11 (30.0)
**AML**	6.08 (0.52-70.89)	0.150	12 (28.9)
**CLO**	6.25 (2.05- 19.10)	**0.001***	13 (22.0)
**DEX**	9.01 (1.30-62.67)	**0.026***	14 (15.8)

Note: presence of (*) and texts in red indicates intervention is statistically significant. Abbreviations: AML, amlexanox paste; AV, aloe vera; CUR, curcumin gel; PDT, photodynamic therapy; PLA, placebo; PUR, purslane; TAC, tacrolimus; FLU, flucinonide acetonide; CLO, clobetasol propionate; PIM, pimecrolimus; BET, betamethasone; TA, triamcinolone acetonide; DEX, dexamethasone; CYC, cyclosporine.

Supplementary Table S10. Results of network meta-analysis: Clinical Resolution of Oral Lichen Planus (subgroup analysis) with SUCRA ranking.

**Interventions**	**Clinical Resolution (Subgroup Analysis)**		
	**Relative Risk (95% CI)**	**P-value**	**SUCRA rank (Score)**
**TAC**	5.40 (1.48- 19.67)	**0.011***	1 (83.1)
**FLU**	9.00 (0.41- 198.97)	0.164	2 (79.2)
**PDT**	3.55 (0.84- 14.94)	0.085	3 (66.8)
**PIM**	2.74 (0.89- 8.37)	0.078	4 (58.1)
**CLO**	2.75 (0.92- 8.22)	0.069	5 (57.2)
**TA**	2.72 (0.82- 9.07)	0.104	6 (56.5)
**CUR**	2.72 (0.11- 66.74)	0.540	7 (55.2)
**AV**	2.18 (0.55- 8.67)	0.268	8 (50.1)
**DEX**	1.18 (0.03- 44.83)	0.928	9 (36.8)
**AML**	1.06 (0.02- 53.30)	0.975	10 (35.6)
**BET**	1.34 (0.43- 4.17)	0.618	11 (33.1)
**PLA**	Reference		12 (20.4)
**CYC**	0.72 (0.12- 4.36)	0.724	13 (17.8)

Note: presence of (*) and texts in red indicates intervention is statistically significant. Abbreviations: AML, amlexanox paste; AV, aloe vera; CUR, curcumin gel; PDT, photodynamic therapy; PLA, placebo; PUR, purslane; TAC, tacrolimus; FLU, flucinonide acetonide; CLO, clobetasol propionate; PIM, pimecrolimus; BET, betamethasone; TA, triamcinolone acetonide; DEX, dexamethasone; CYC, cyclosporine.

Supplementary Table S11. Results of network meta-analysis: Clinical Score (subgroup analysis) with SUCRA ranking.

Interventions	Clinical Score (Subgroup Analysis)		
	Mean Difference (95% CI)	P-value	SUCRA rank (Score)
**PLA**	Reference		1 (78.9)
**BET**	-0.52 (-3.35- 2.32)	0.720	2 (62.6)
**HA**	-0.80 (-2.53- 0.93)	0.364	3 (56.3)
**PIM**	-0.86 (-3.30- 1.58)	0.492	4 (55.2)
**DEX**	-0.07 (-62.08- 61.95)	0.998	5 (54.5)
**CYC**	-0.89 (-2.66- 0.89)	0.329	6 (54.3)
**AML**	0.06 (-61.95- 62.07)	0.998	7 (54.2)
**CLO**	-1.02 (-2.59- 0.54)	0.199	8 (52.4)
**FLU**	-1.11 (-3.21- 1.00)	0.303	9 (48.7)
**AV**	-1.08 (-2.56- 0.39)	0.150	10 (47.9)
**TA**	-1.12 (-3.05- 0.81)	0.256	11 (46.6)
**TAC**	-1.25 (-3.03- 0.53)	0.170	12 (43.2)
**CUR**	-1.46 (-4.29- 1.38)	0.314	13 (39.3)
**PDT**	-5.92 (-8.76- (-3.09))	**0.000***	14 (6.1)

Note: presence of (*) and texts in red indicates intervention is statistically significant. Abbreviations: AML, amlexanox paste; AV, aloe vera; CUR, curcumin gel; PDT, photodynamic therapy; PLA, placebo; PUR, purslane; TAC, tacrolimus; FLU, flucinonide acetonide; CLO, clobetasol propionate; PIM, pimecrolimus; BET, betamethasone; TA, triamcinolone acetonide; DEX, dexamethasone; CYC, cyclosporine; HA, hyaluronic acid.

Supplementary Table S12. Results of network meta-analysis: Pain Score (subgroup analysis) with SUCRA ranking.

**Interventions**	**Pain Score (Subgroup Analysis)**		
	**Mean Difference (95% CI)**	**P-value**	**SUCRA rank (Score)**
**PDT**	-1.90 (-3.07- (-0.73))	**0.001***	1 (92.1)
**TAC**	-1.61 (-2.78- (-0.45))	**0.007***	2 (86.3)
**CLO**	-0.93 (-1.88- 0.02)	0.055	3 (59.5)
**CYC**	-0.87 (-1.93- 0.18)	0.106	4 (59.0)
**TA**	-0.75 (-1.79- 0.29)	0.159	5 (53.7)
**HA**	-0.64 (-2.38- 1.10)	0.469	6 (46.1)
**PRED**	-0.59 (-2.77- 1.59)	0.596	7 (44.3)
**PIM**	-0.42 (-1.37- 0.53)	0.382	8 (37.1)
**CUR**	-0.43 (-2.15- 1.28)	0.622	9 (35.7)
**AV**	0.07 (-1.05- 1.18)	0.907	10 (18.5)
**PLA**	Reference		11 (17.7)

Note: presence of (*) and texts in red indicates intervention is statistically significant. Abbreviations: AML, amlexanox paste; AV, aloe vera; CUR, curcumin gel; PDT, photodynamic therapy; PLA, placebo; PUR, purslane; TAC, tacrolimus; FLU, flucinonide acetonide; CLO, clobetasol propionate; PIM, pimecrolimus; BET, betamethasone; TA, triamcinolone acetonide; DEX, dexamethasone; CYC, cyclosporine; HA, hyaluronic acid; PRED; prednisolone.

Supplementary Table S13. GRADE Assessment for Quality of Evidence

Certainty assessment							№ of patients		Effect		Certainty	Importance
№ of studies	Study design	Risk of bias	Inconsistency	Indirectness	Imprecision	Other considerations	different interventions	placebo/ interventions	Relative (95% CI)	Absolute (95% CI)		
**clinical improvement of oral lichen planus in terms of extension and severity (follow-up: mean 4 weeks; assessed with: signs of improvement)**												
28	randomised trials	serious[a]	not serious	not serious	not serious	none	436	357	-	see comment	⨁⨁⨁◯ Moderate	CRITICAL
**reduction in pain score (follow-up: mean 4 weeks; assessed with: VAS score)**												
20	randomised trials	serious	not serious	not serious	not serious	none			-	not estimable	⨁⨁⨁◯ Moderate	IMPORTANT
**reduction in clinical score (follow-up: mean 4 weeks; assessed with: mean)**												
28	randomised trials	serious	not serious	not serious	not serious	none			-	not estimable	⨁⨁⨁◯ Moderate	IMPORTANT
**clinical resolution (follow-up: mean 4 weeks; assessed with: Thongprasom scale)**												
20	randomised trials	serious	not serious	not serious	not serious	none	167	113	-	see comment	⨁⨁⨁◯ Moderate	CRITICAL
**adverse effects caused by intervention (follow-up: mean 4 weeks)**												
30	randomised trials	serious	not serious	not serious	not serious	none	128	47	-	see comment	⨁⨁⨁◯ Moderate	CRITICAL

Explanations

a. Eventhough majority of the included articles have Low or unclear risk of bias, 16 articles have high risk of bias. Hence, quality of evidence may be moderate

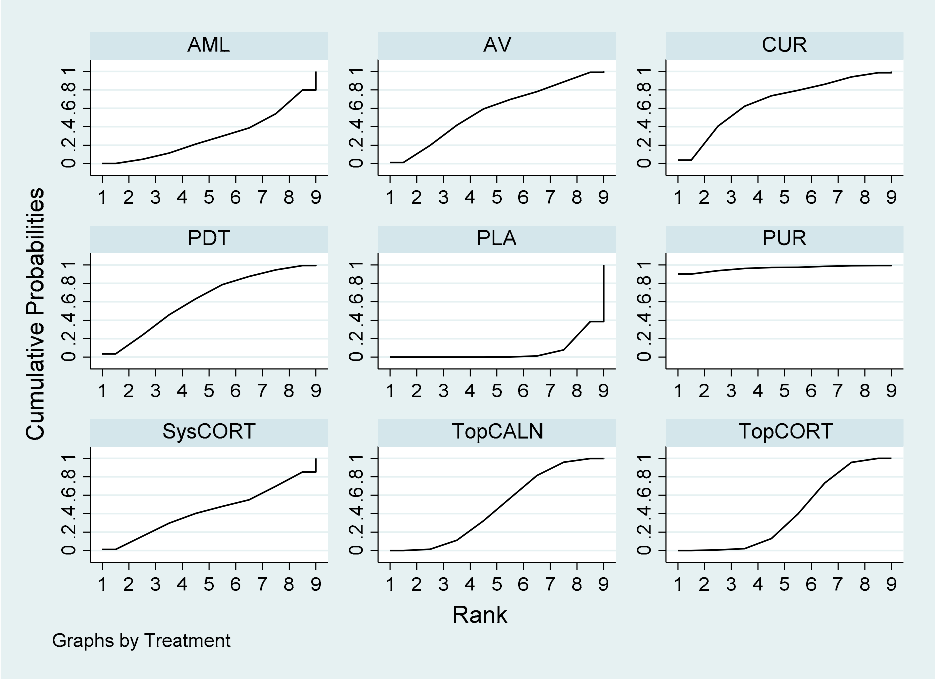

Supplementary Figure S1. SUCRA ranking curve in Clinical Improvement of OLP

Abbreviations: AML, amlexanox paste; AV, aloe vera; CUR, curcumin gel; PDT, photodynamic therapy; PLA, placebo; PUR, purslane; SysCORT, systemic corticosteroid; TopCALN, topical calcineurin; TopCALNcoSysCORT, topical calcineurin combined with systemic corticosteroid; TopCORT, topical corticosteroid

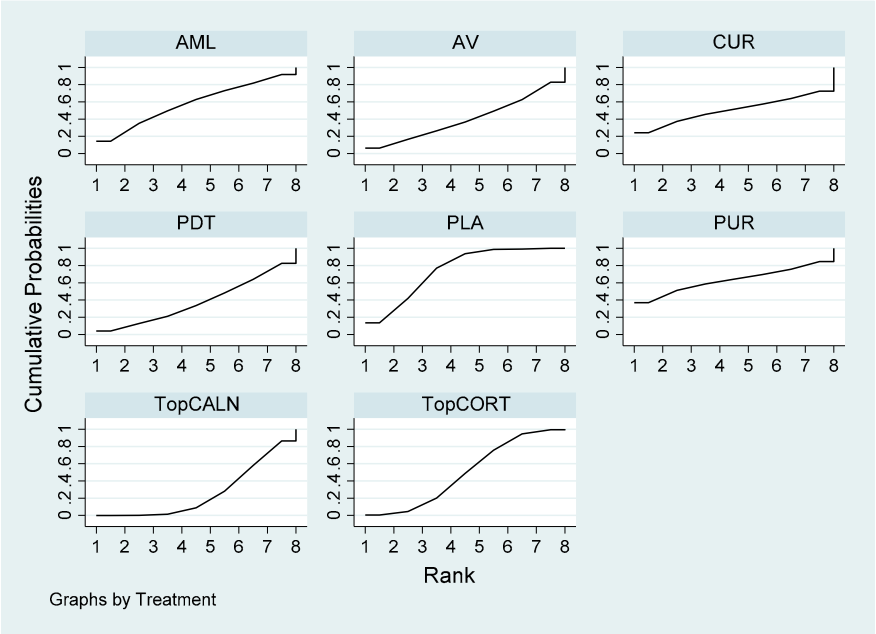

Supplementary Figure S2. SUCRA ranking curve of Adverse Effects

Abbreviations: AML, amlexanox paste; AV, aloe vera; CUR, curcumin gel; PDT, photodynamic therapy; PLA, placebo; PUR, purslane; TopCALN, topical calcineurin; TopCORT, topical corticosteroid

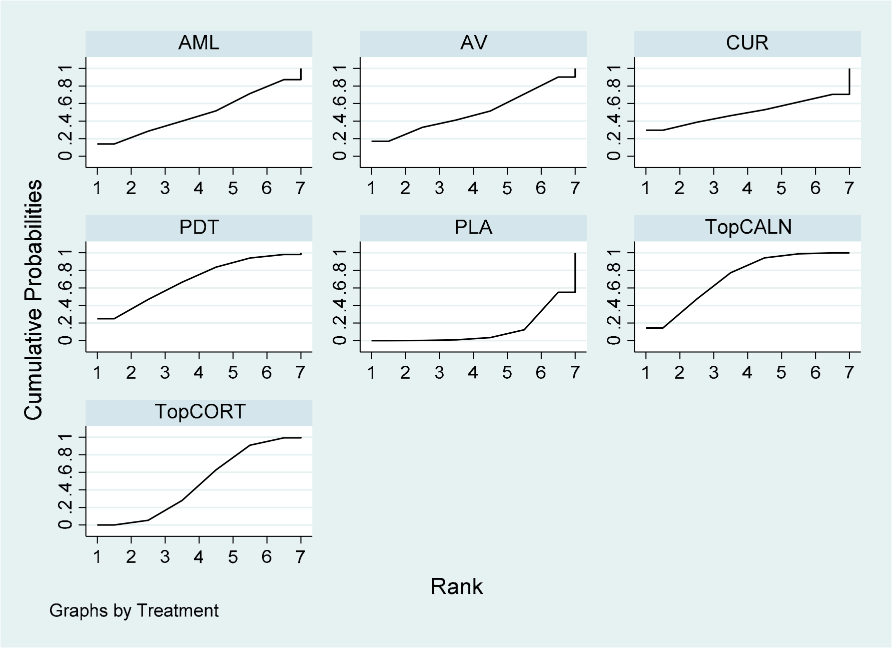

Supplementary Figure S3: SUCRA ranking curve of Clinical Resolution or Oral Lichen Planus. Abbreviations: AML, amlexanox paste; AV, aloe vera; CUR, curcumin gel; PDT, photodynamic therapy; PLA, placebo; PUR, purslane; TopCALN, topical calcineurin; TopCORT, topical corticosteroid

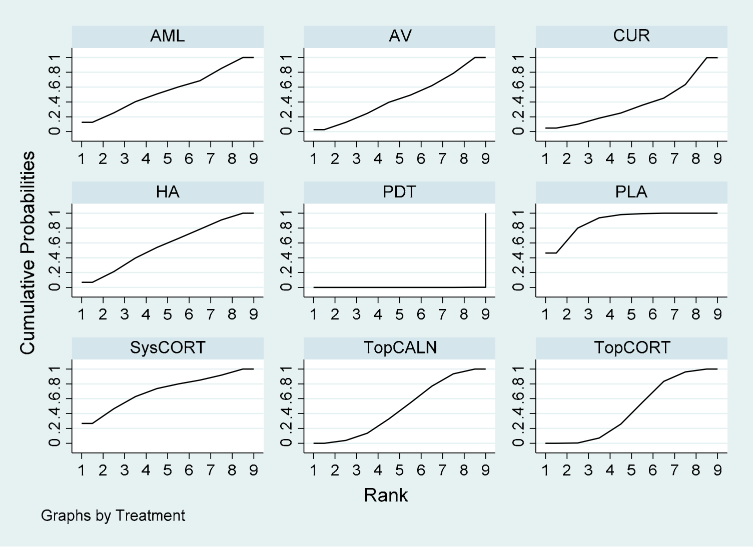

Supplementary Figure S4. SUCRA ranking curve of Clinical Score. Abbreviations: AML, amlexanox paste; AV, aloe vera; CUR, curcumin gel; PDT, photodynamic therapy; PLA, placebo; PUR, purslane; SysCORT, systemic corticosteroid; TopCALN, topical calcineurin; TopCORT, topical corticosteroid; HA, hyaluronic acid

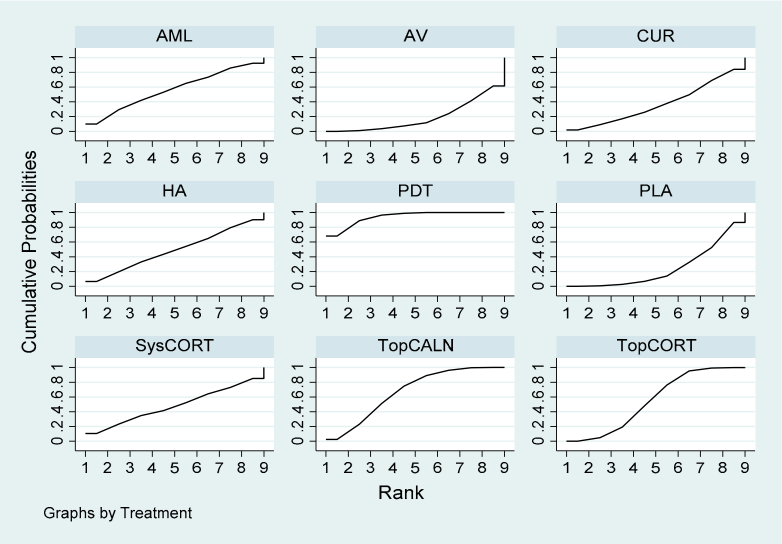

Supplementary Figure S5. SUCRA ranking curve of Pain Score. Abbreviations: AML, amlexanox paste; AV, aloe vera; CUR, curcumin gel; PDT, photodynamic therapy; PLA, placebo; PUR, purslane; SysCORT, systemic corticosteroid; TopCALN, topical calcineurin; TopCORT, topical corticosteroid; HA, hyaluronic acid

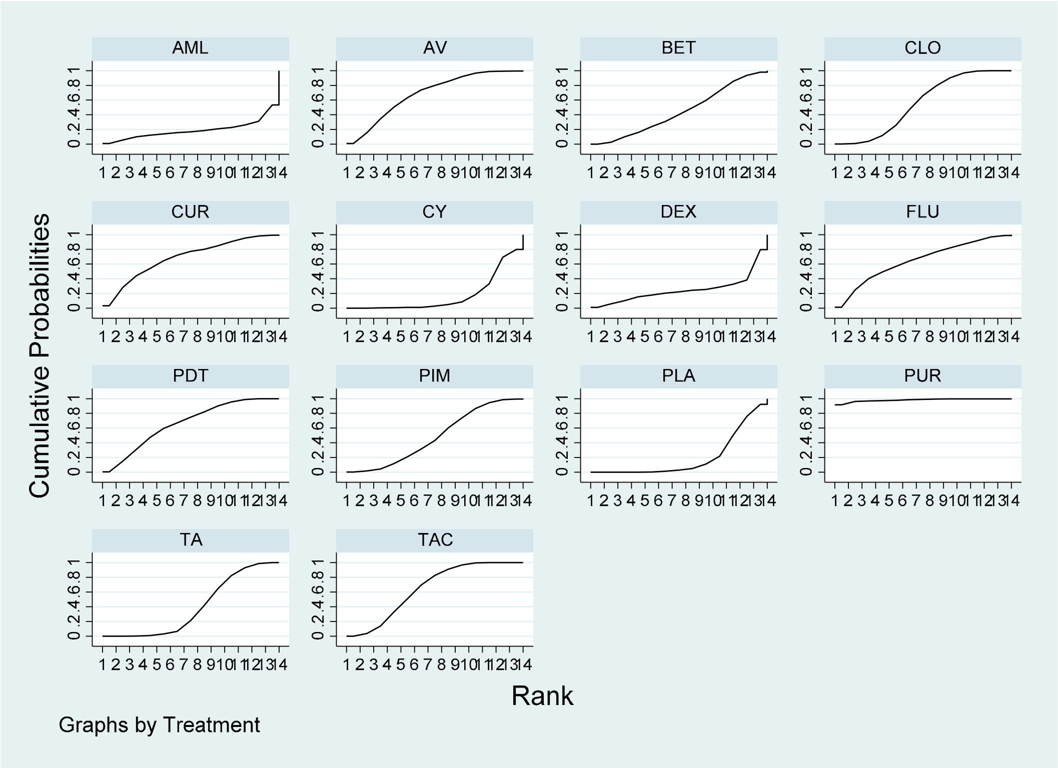

Supplementary Figure S6. SUCRA ranking curve in Clinical Improvement of OL (subgroup). Abbreviations: AML, amlexanox paste; AV, aloe vera; CUR, curcumin gel; PDT, photodynamic therapy; PLA, placebo; PUR, purslane; TAC, tacrolimus; FLU, flucinonide acetonide; CLO, clobetasol propionate; PIM, pimecrolimus; BET, betamethasone; TA, triamcinolone acetonide; DEX, dexamethasone; CYC, cyclosporine.

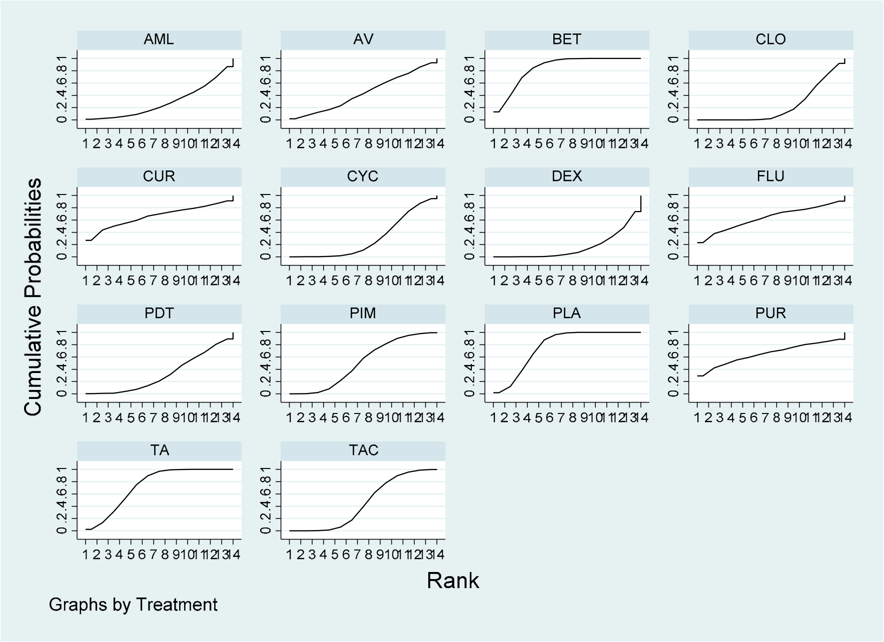

Supplementary Figure S7. SUCRA ranking curve of Adverse Effects. Abbreviations: AML, amlexanox paste; AV, aloe vera; CUR, curcumin gel; PDT, photodynamic therapy; PLA, placebo; PUR, purslane; TAC, tacrolimus; FLU, flucinonide acetonide; CLO, clobetasol propionate; PIM, pimecrolimus; BET, betamethasone; TA, triamcinolone acetonide; DEX, dexamethasone; CYC, cyclosporine.

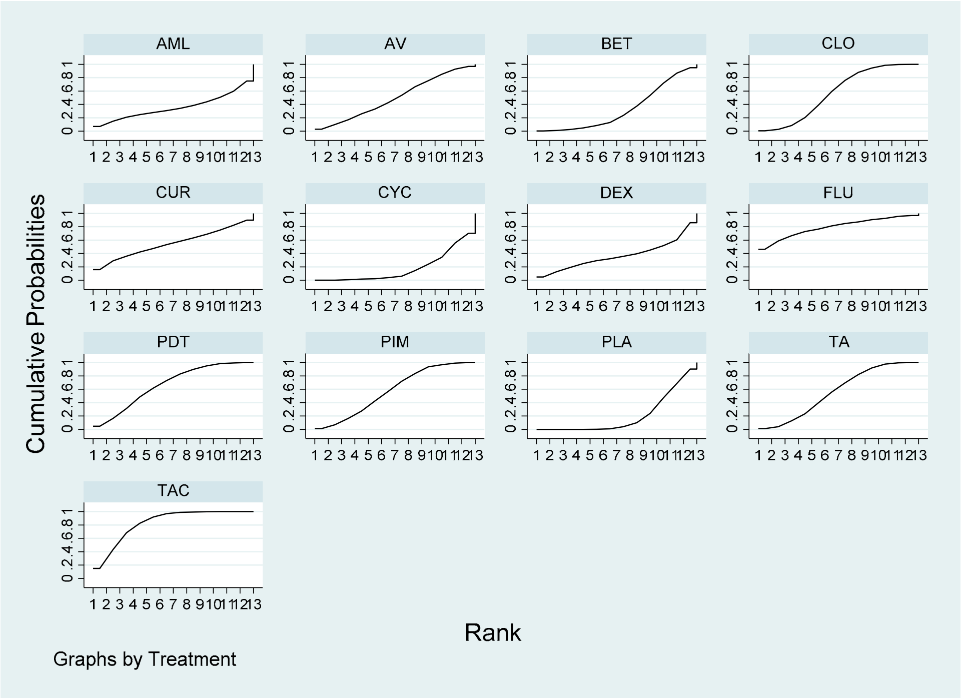

Supplementary Figure S8: SUCRA ranking curve of Clinical Resolution or Oral Lichen Planus. Abbreviations: AML, amlexanox paste; AV, aloe vera; CUR, curcumin gel; PDT, photodynamic therapy; PLA, placebo; PUR, purslane; TAC, tacrolimus; FLU, flucinonide acetonide; CLO, clobetasol propionate; PIM, pimecrolimus; BET, betamethasone; TA, triamcinolone acetonide; DEX, dexamethasone; CYC, cyclosporine.

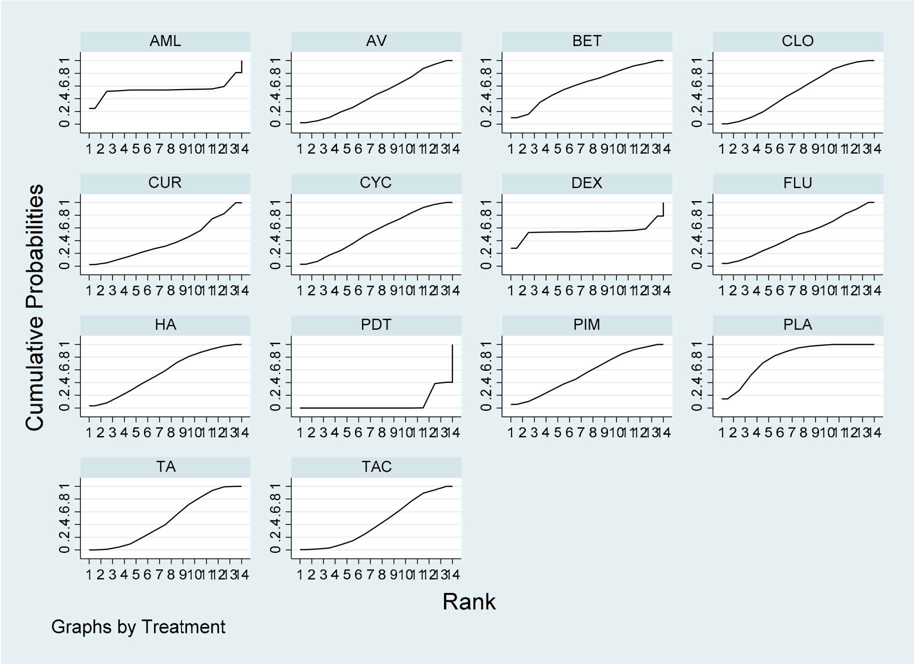

Supplementary Figure S9: SUCRA ranking curve of Clinical Score

Abbreviations: AML, amlexanox paste; AV, aloe vera; CUR, curcumin gel; PDT, photodynamic therapy; PLA, placebo; PUR, purslane; TAC, tacrolimus; FLU, flucinonide acetonide; CLO, clobetasol propionate; PIM, pimecrolimus; BET, betamethasone; TA, triamcinolone acetonide; DEX, dexamethasone; CYC, cyclosporine; HA, hyaluronic acid

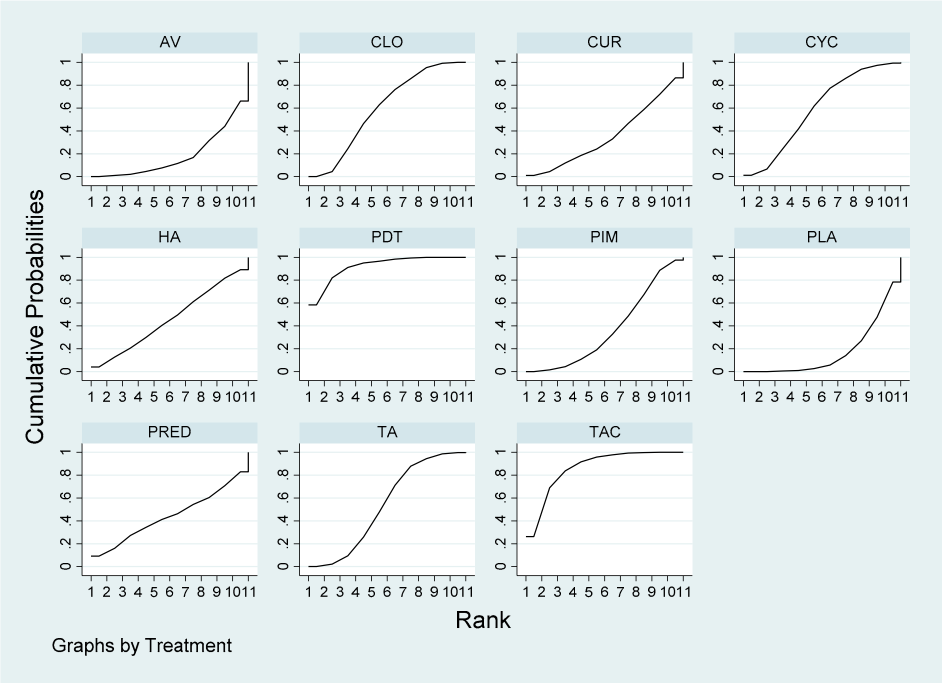

Supplementary Figure S10. SUCRA ranking curve of Pain Score

Abbreviations: AML, amlexanox paste; AV, aloe vera; CUR, curcumin gel; PDT, photodynamic therapy; PLA, placebo; PUR, purslane; TAC, tacrolimus; FLU, flucinonide acetonide; CLO, clobetasol propionate; PIM, pimecrolimus; BET, betamethasone; TA, triamcinolone acetonide; DEX, dexamethasone; CYC, cyclosporine; HA, hyaluronic acid; PRED; prednisolone

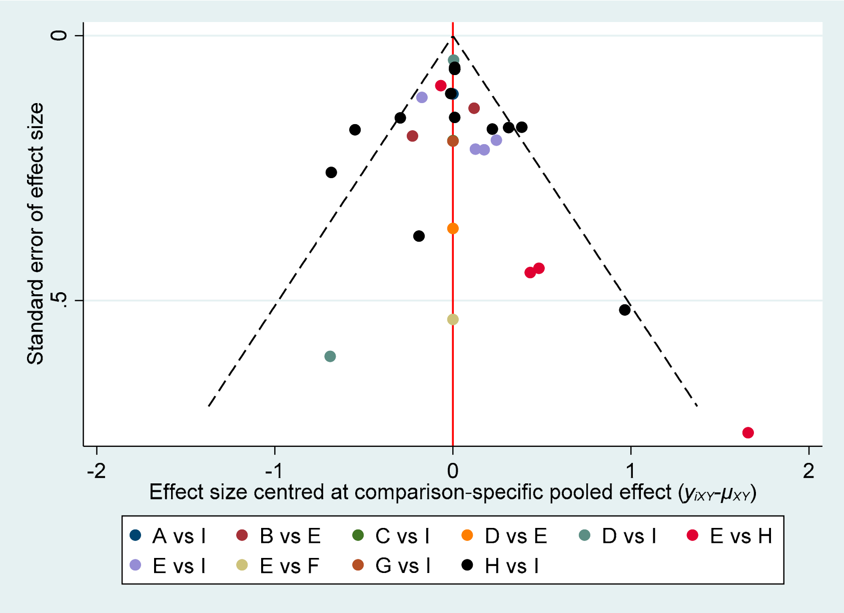

Supplementary Figure S11. Comparison-adjusted funnel plot for Clinical Improvement of Oral Lichen Planus

Abbreviations: A= amlexanox paste; B= aloe vera; C= curcumin gel; D= photodynamic therapy; E= placebo; F= purslane; G= systemic corticosteroid; H= topical calcineurin; I= combination of topical calcineurin and systemic corticosteroid; J= topical corticosteroid

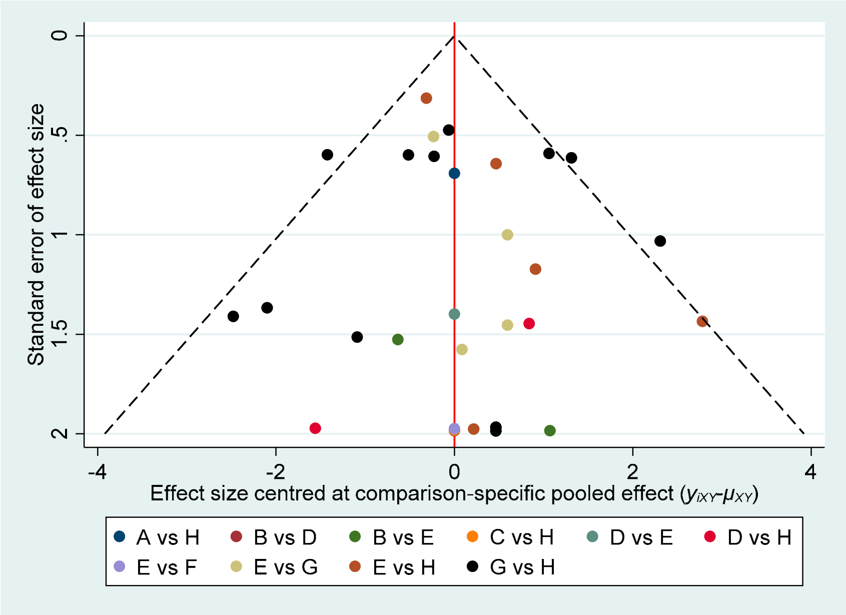

Supplementary Figure S12: Comparison-adjusted funnel plot for Adverse Effects

Abbreviations: A= amlexanox paste; B= aloe vera; C= curcumin gel; D= photodynamic therapy; E= placebo; F= purslane; G= topical calcineurin; H= topical corticosteroid

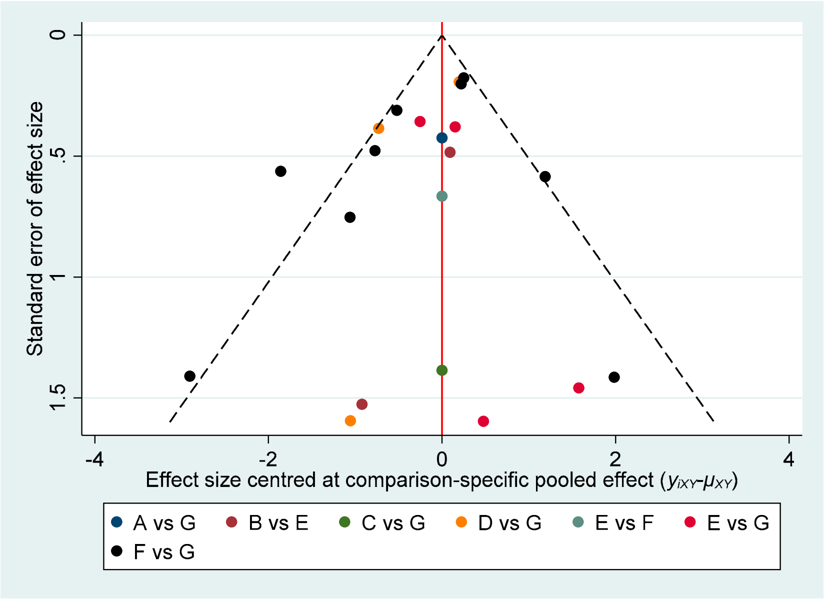

Supplementary Figure S13: Comparison-adjusted funnel plot for Clinical Resolutions
Abbreviations: A= amlexanox paste; B= aloe vera; C= curcumin gel; D= photodynamic therapy; E= placebo; F= purslane; G= systemic corticosteroid; H= topical calcineurin; I= combination of topical calcineurin and systemic corticosteroid; J= topical corticosteroid

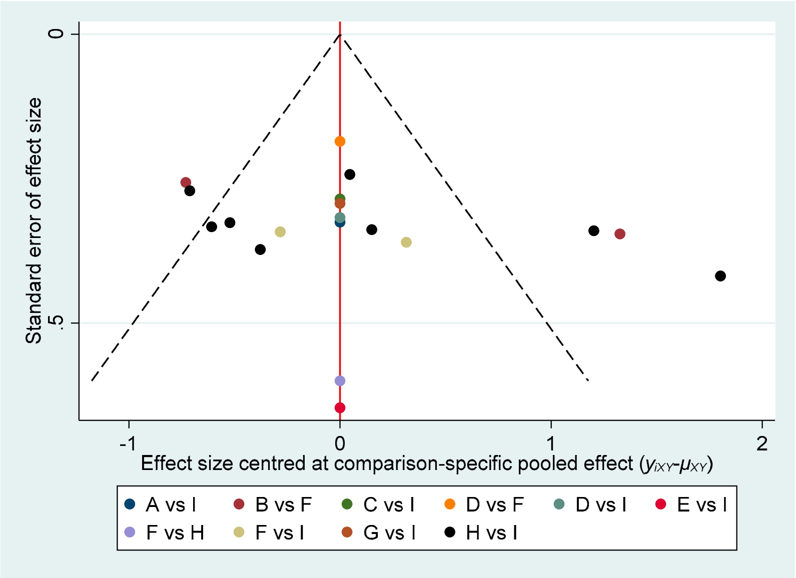

Supplementary Figure S14. Comparison-adjusted funnel plot for Clinical Scores
Abbreviations: A= amlexanox paste; B= aloe vera; C= curcumin gel; D= photodynamic therapy; E= placebo; F= purslane; G= systemic corticosteroid; H= topical calcineurin; I= combination of topical calcineurin and systemic corticosteroid; J= topical corticosteroid

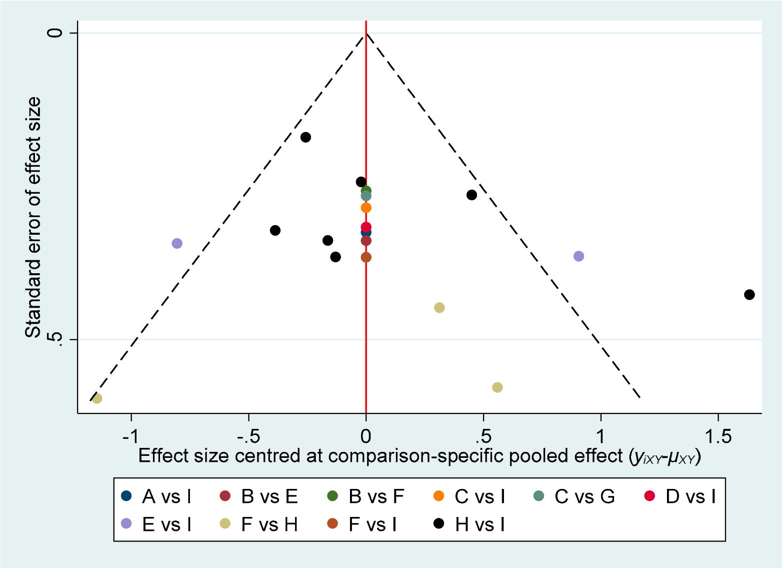

Supplementary Figure S15: Comparison-adjusted funnel plot for Pain Scores

Abbreviations: A= amlexanox paste; B= aloe vera; C= curcumin gel; D= photodynamic therapy; E= placebo; F= purslane; G= systemic corticosteroid; H= topical calcineurin; I= combination of topical calcineurin and systemic corticosteroid; J= topical corticosteroid

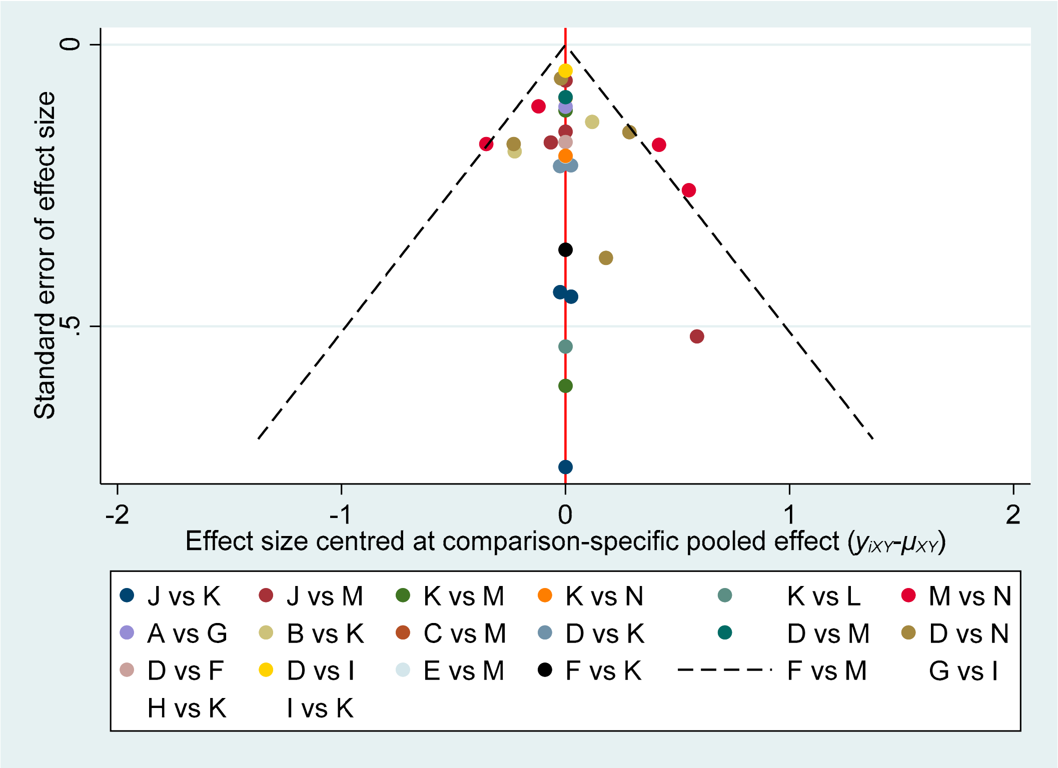

Supplementary Figure S16: Comparison-adjusted funnel plot for Clinical Improvement of Oral Lichen Planus

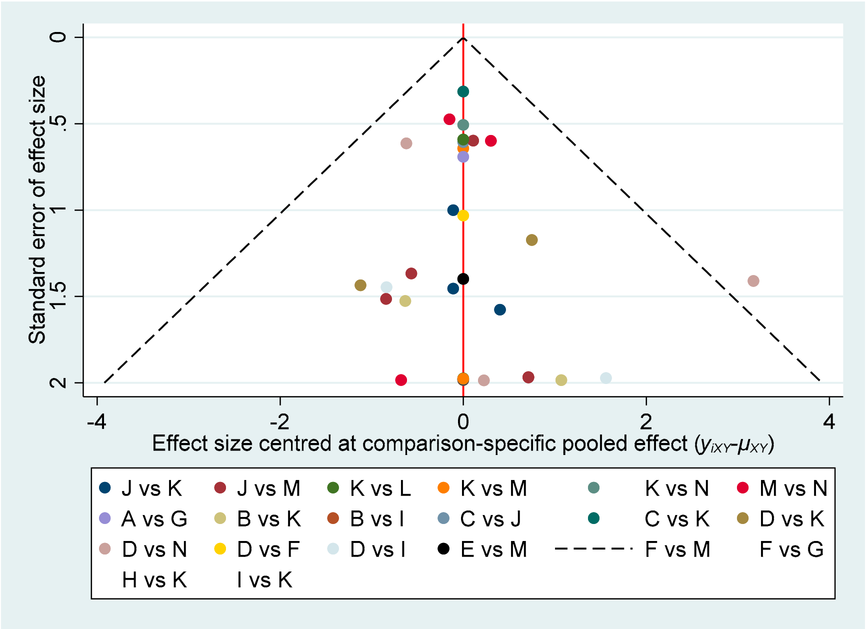

Supplementary Figure S17. Comparison-adjusted funnel plot for Adverse effects of Oral Lichen Planus

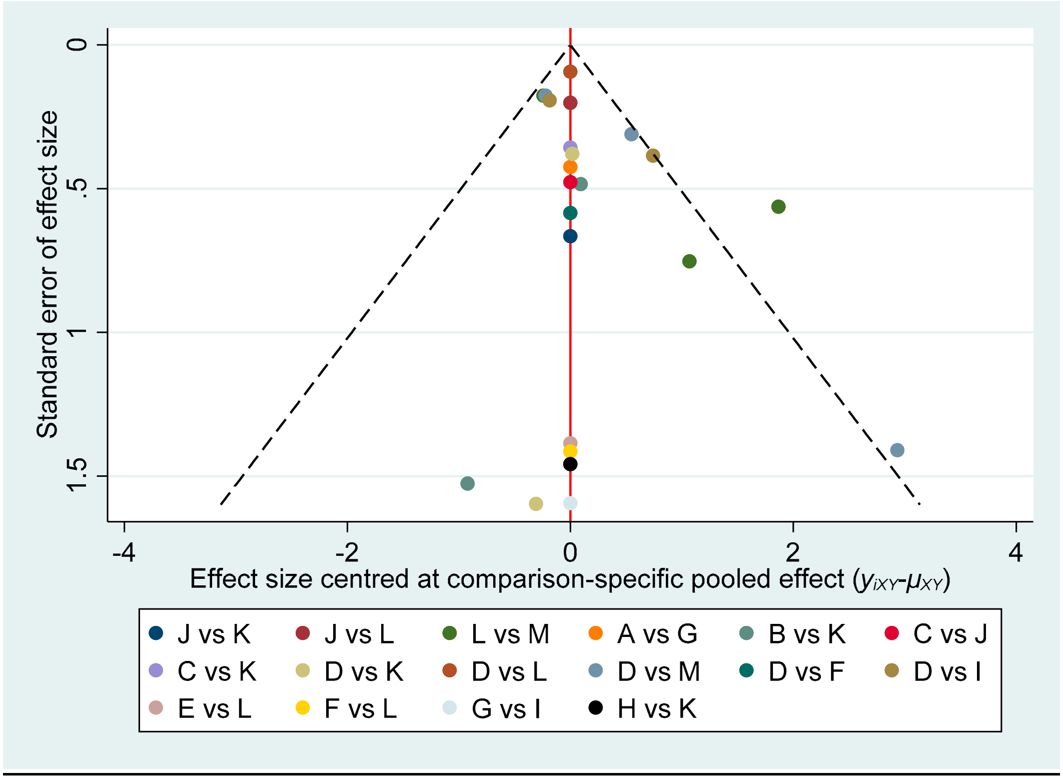

Supplementary Figure S18. Comparison-adjusted funnel plot for Clinical Resolution of Oral Lichen Planus

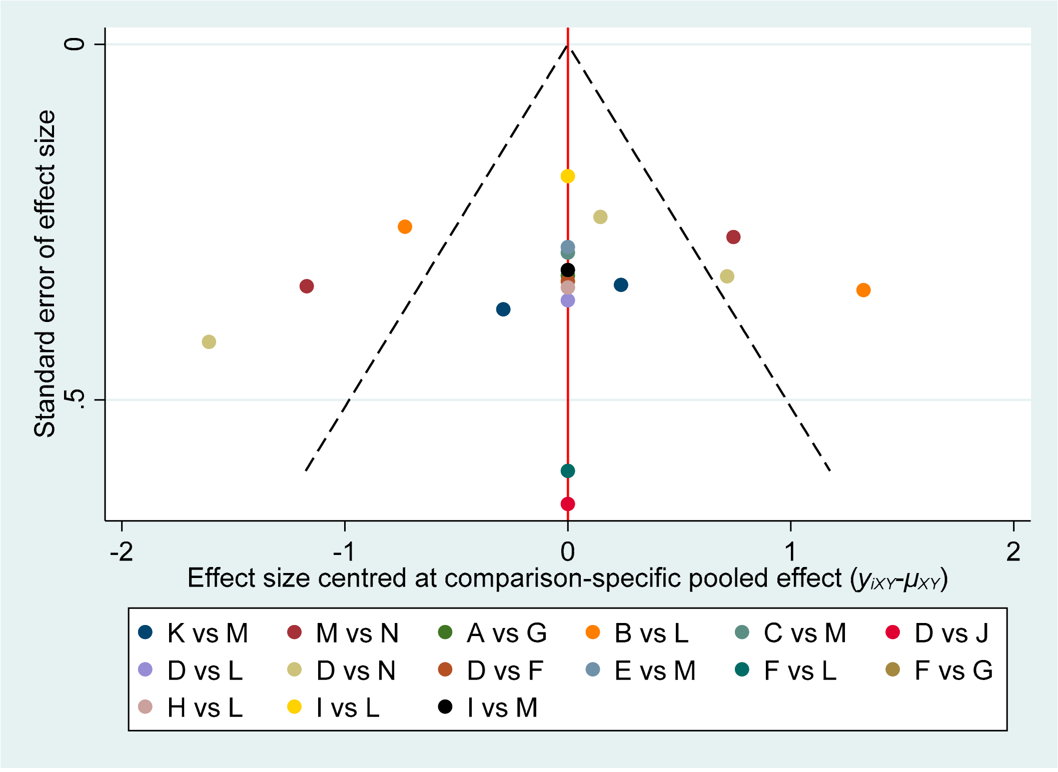

Supplementary Figure S19. Comparison-adjusted funnel plot for Clinical Score of Oral Lichen Planus

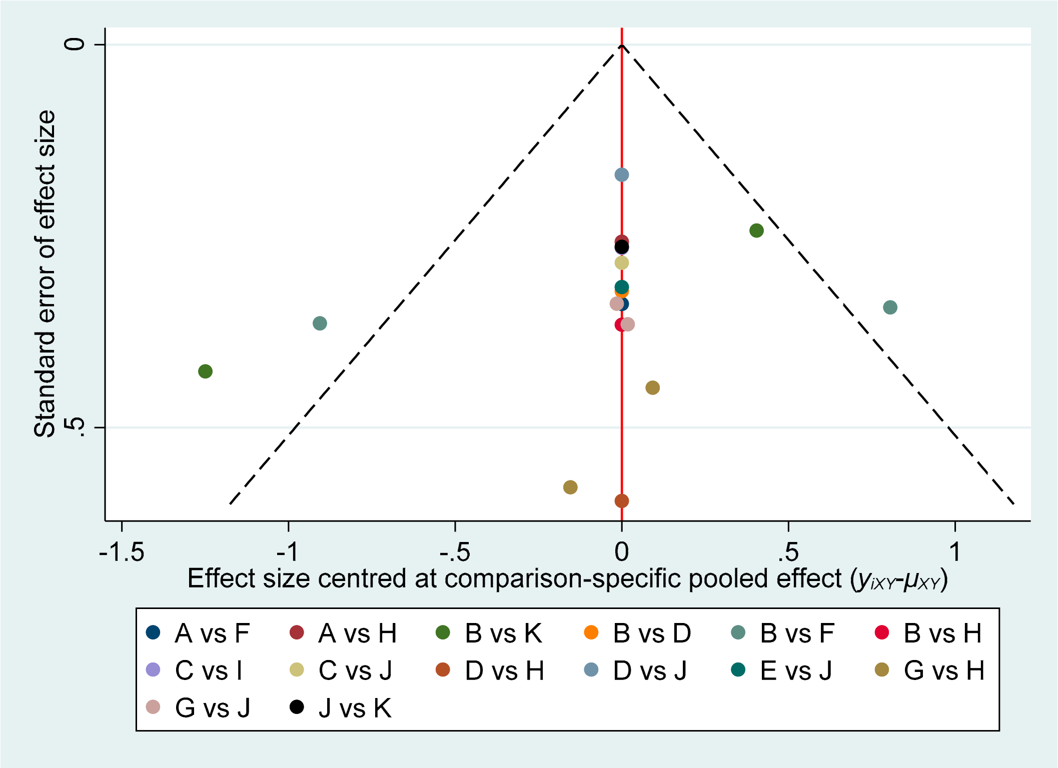

Supplementary Figure S20. Comparison-adjusted funnel plot for Pain Score of Oral Lichen Planus
